# The glycolytic enzyme PGK1 phosphorylates MORC2 to Confer radioresistance in pancreatic ductal adenocarcinoma

**DOI:** 10.1038/s41419-025-08177-9

**Published:** 2025-11-10

**Authors:** Yingying Tong, Xin Liu, Qian Liu, Jing Wang, Yaoxian Xiang, Kangjie Wang, Zezhou Zhao, Ke Zhu, Lijun Yang, Li Wang, Dong Guo, Zhimin Lu, Dong Yan

**Affiliations:** 1 Cancer Center, Beijing Luhe Hospital, Capital Medical University, Beijing, China; 2 Zhejiang Provincial Key Laboratory of Pancreatic Disease, the First Affiliated Hospital, Zhejiang Key Laboratory of Frontier Medical Research on Cancer Metabolism, Institute of Translational Medicine, Zhejiang University School of Medicine, Hangzhou, Zhejiang China; 3 Institute of Fundamental and Transdisciplinary Research, Cancer Center, Zhejiang University, Hangzhou, Zhejiang China

**Keywords:** Post-translational modifications, Pancreatic cancer, DNA damage and repair

## Abstract

Most pancreatic cancer patients exhibit inherent resistance to radiation therapy, and the molecular mechanisms remain poorly understood. Here, we demonstrate that phosphoglycerate kinase 1 (PGK1), a key ATP-producing glycolytic enzyme, plays a critical role in pancreatic ductal adenocarcinoma (PDAC) radioresistance. In response to ionizing radiation (IR), casein kinase 2 (CK2) is activated and phosphorylates PGK1 at S256. Phosphorylated PGK1 interacts with microrchidia CW-type zinc finger 2 (MORC2). Importantly, PGK1 functions as a protein kinase and phosphorylates MORC2 at S711, thereby enhancing the DNA-dependent ATPase activity of MORC2 to facilitate chromatin remodeling and DNA repair. Disruption of CK2-mediated PGK1 phosphorylation or PGK1-dependent MORC2 phosphorylation sensitizes PDAC cells and mouse tumors to IR. Clinically, the levels of PGK1 pS256 and MORC2 pS711, which are mutually correlated, are positively associated with poor survival in radiotherapy-treated PDAC specimens. These findings highlight the critical role of the nonmetabolic functions of PGK1 in DNA damage repair and PDAC radioresistance.

## Introduction

Pancreatic ductal adenocarcinoma (PDAC), the predominant form of pancreatic cancer, is a highly malignant tumor of the digestive system with a dismal 5-year overall survival rate, severely limiting therapeutic advancements [1]. The lethality of PDAC is primarily due to late-stage diagnosis, often post-metastasis, necessitating urgent investigation to improve patient outcomes. Currently, chemotherapy and surgery are the main treatment modalities for PDAC. Most research has focused on understanding tumor progression mechanisms [2–4], developing novel therapeutic targets, and addressing chemotherapy and immunotherapy resistance [5–7], with less emphasis on radiotherapy. Recent randomized clinical trials have shown that neoadjuvant chemoradiation combined with adjuvant chemotherapy improves R0 resection rates, reduces lymph-node metastasis, lowers locoregional recurrence, and significantly enhances 5-year overall survival compared to upfront surgery with adjuvant therapy [8]. This underscores the importance of radiotherapy in PDAC treatment. However, the response to radiotherapy varies significantly among PDAC patients, and the underlying mechanisms remain poorly understood. Thus, elucidating the molecular basis of radioresistance and identifying novel targets are crucial for enhancing radiotherapy efficacy in PDAC patients.

Phosphoglycerate kinase 1 (PGK1) is upregulated in various human cancers, including PDAC [9], breast cancer [10], radioresistant astrocytoma [11], and multidrug-resistant ovarian cancer cells [12]. As the first ATP-generating enzyme in glycolysis, PGK1 catalyzes the conversion of 1,3-biphosphoglycerate (1,3-BPG) to 3-phosphoglycerate (3-PG) and ATP [13], coordinating energy production with biosynthesis. Beyond its glycolytic role, PGK1 functions as a protein kinase (Fig. S1A). For example, PGK1 phosphorylates pyruvate dehydrogenase kinase 1 (PDHK1) at T338 and Beclin1 at S30, thereby regulating mitochondrial metabolism and autophagy [14, 15]. Additionally, casein kinase 2 (CK2) phosphorylates nuclear PGK1 at S256, facilitating its interaction with CDC7, which promotes the recruitment of DNA helicase to replication origins, DNA replication, cell proliferation, and tumorigenesis [16]. However, PGK1 is involvement in other critical cellular activities as a protein kinase remains to be fully explored.

ATP-dependent chromatin remodeling enzymes are pivotal in DNA-templated reactions in eukaryotes, with deregulation implicated in various cancers [17]. Microrchidia CW-type zinc finger 2 (MORC2), a member of the MORC ATPase superfamily, is one such enzyme [18, 19]. MORC2 contains a CW-type zinc finger (CW-ZF) domain and an N-terminal ATPase module, essential for epigenetic gene silencing [20, 21] and DNA damage repair via chromatin remodeling [22]. The CW-ZF domain enhances DNA binding and protein-protein interactions [23–25]. Recent studies indicate that MORC2 is phosphorylated in response to DNA damage, facilitating ATPase-dependent chromatin remodeling and efficient DNA repair [26]. However, the precise mechanisms by which MORC2 regulates DNA repair signaling are not fully understood.

In this study, we identify PGK1 as highly expressed in PDAC specimens, contributing to PDAC radioresistance. IR activates CK2, which phosphorylates PGK1 at S256. Phosphorylated PGK1 interacts with MORC2, phosphorylating it at S711. This phosphorylation enhances MORC2’s DNA-dependent ATPase activity, promoting chromatin remodeling and DNA repair. Disrupting CK2-mediated PGK1 phosphorylation or PGK1-dependent MORC2 phosphorylation sensitizes PDAC cells and tumors to IR. Clinical evidence shows that the levels of PGK1 pS256 and MORC2 pS711 are mutually correlated and are positively associated with poor survival in radiotherapy-treated PDAC specimens. These findings highlight the critical role of PGK1’s nonmetabolic functions in DNA damage repair and PDAC radioresistance.

## Materials and methods

### Cell culture

293T, HPDE, and PANC-1 cells were maintained in DMEM supplemented with 10% fetal bovine serum (FBS). AsPC-1, BxPC-3 and SW1990 cells were maintained in RPMI 1640 medium supplemented with 10%. All of the cell lines were obtained from ATCC. Prior to use, all cell lines were authenticated and tested to ensure the absence of mycoplasma contamination. Additionally, none of the cell lines used in this study were listed in the database of commonly misidentified cell lines maintained by the National Center for Biotechnology Information (NCBI) and the International Cell Line Authentication Committee (ICLAC).

### DNA constructs and mutagenesis

PCR-amplified human PGK1and MORC2 were cloned into pLenti-puro-3 × Flag, pLVX-puro-V5, pET28A, or pGEX-4T-1 vector. pLenti-puro-3 × Flag PGK1 S256A, MORC2 S711A, MORC2 S711D, MORC2 D68A, pGEX-4T-1 PGK1 S256A, T378P, and pET28A MORC2 S711A were generated using the QuikChange site-directed mutagenesis kit (Vazyme, China). All mutants were verified by full-length cDNA sequencing. sgRNA was constructed via ligation of an oligonucleotide targeting human PGK1 and MORC2 into an Bbsl-digested LentiCRISPRv2 vector. All sequences are listed in Table S1.

### Immunoprecipitation and immunoblotting analysis

Proteins were extracted from cultured cells using RIPA buffer or 1% NP-40 buffer (50 mM Tris-HCl [pH8.0], 150 mM NaCl, 5 mM EDTA, 1% NP-40, and 10% glycerol) protease inhibitor cocktails and PhosSTOP phosphatase inhibitor cocktails (Roche). For co-immunoprecipitation, the cell lysate supernatant was mixed with indicated antibodies overnight at 4 °C and then with 20 μL Protein A/G Dynabeads or Anti-FLAG M2 Beads for overnight at 4 °C. Proteins were separated by SDS-PAGE gel and transferred to PVDF membranes. After blocking, membranes were probed using corresponding antibodies and then HRP-linked secondary antibodies. Signals were visualized using the ECL method.

### Real-time PCR analyses

Total RNA was extracted from PANC-1 and AsPC-1 cells using TRIzol reagent (Invitrogen) following the manufacturer’s protocol. cDNA was synthesized from 1 μg of RNA using Maxima Reverse Transcriptase (Thermo Fisher Scientific). Real-time PCR was conducted in triplicate on an Applied Biosystems 7900 system, with *GADPH* mRNA as the normalization control. Primer sequences are listed in Supplementary Table S2.

### Mass spectrometry analysis

PGK1-associated proteins from the immunoprecipitation assay and in vitro PGK1-phosphorylated MORC2 were precipitated with acetone overnight at −20 °C and resuspended in 50 mM ammonium bicarbonate buffer containing RapiGest (WatersCorp). The sample was heated to 95 °C for 10 min, cooled, and then digested with 100 ng of sequencing-grade modified trypsin (Promega) at 37 °C overnight. The digested sample was analyzed by a hybrid Q-Exactive mass spectrometer (Thermo Fisher Scientific) for 60 min. MS data were searched against the UniProt database (https://www.uniprot.org/). All peptide matches were filtered based on mass measurement errors and Xcorr and Corr scores, and phosphorylation sites were manually validated.

### In vitro and in vivo lR

For in vitro studies, cells were irradiated at various doses with an XRAD 320 X-ray irradiator (Precision X-ray Inc., USA) at a dose rate of 75 cGy/min. For in vivo studies, mice were anesthetized allowing for local delivery of radiation using an XRAD 320 X-ray irradiator (Precision X-Ray, Inc., CT, USA) at a dose rate of 289.8 cGy/min.

### Tumor xenograft and orthotopic mouse models

The subcutaneous xenograft model of PDAC was established as previously described [27]. Briefly, 1 × 10^6^ PANC-1 cells (in 100 μl of PBS) with or without PGK1 or MORC2 modulation were subcutaneously injected into the flanks of 6-week-old male athymic nude mice (6 mice per group). For orthotopic models, 1 × 10^6^ PANC-1 cells (50 μl PBS) with indicated PGK1 or MORC2 expression were injected into the pancreatic tail through a 1.5 cm flank incision, followed by wound closure (6 mice per group).

Tumor growth was monitored every 3 days (caliper for subcutaneous, ultrasound for orthotopic). When volumes reached 80–100 mm^3^, mice were randomized for treatment. Radiotherapy (2 Gy/fraction) was administered as shown in Fig. 6B. Mice were sacrificed 24 days post-implantation, and tumors were harvested, weighed, fixed, and paraffin-embedded.

### Clonogenic formation assay

PANC-1 and AsPC-1 cells were seeded in triplicate in different numbers according to the irradiation dose they had accepted. 24 h later, cells were irradiated with different doses of X-ray (0 Gy, 2 Gy, 4 Gy, 6 Gy and 8 Gy with 500, 500, 1000, 2000, and 5000 cells per plate, respectively) and then incubated for 2 weeks. The colonies were stained with crystal violet, and the numbers of colonies with 50 or more cells were counted. The surviving fraction was calculated as the ratio of the plating efficiency of the treated cells to that of control cells.

### Immunofluorescence analyses

Immunofluorescent staining of γ-H2AX was performed following published instructions [28]. In brief, cells were incubated with complete medium for 24 h prior to IR treatment. At indicated time points, cells were washed with cold PBS. Coverslips were then blocked by incubation in 5% BSA for 30 min. Blocked coverslips were then incubated with antibodies detecting γ-H2AX (at a 1 : 250 dilution), fluorescence dye-conjugated secondary antibodies, and DAPI according to standard protocols. Immunofluorescence microscopic images of the cells were obtained and viewed using confocal microscopy (Leica, Germany). For quantification, cells containing more than 5 foci were determined as positive.

### Comet assay

Single cell gel electrophoresis (Alkaline) was performed according to manufacturer’s instructions. Briefly, cells were irradiated at 4 Gy and were collected at 0, 12, and 24 h after IR, followed by washing and resuspension in cold PBS. 2 × 10^4^ cells were prepared for each assay using the Comet Assay Electrophoresis system (AmyJet Scientific Inc). The samples were then analyzed by fluorescence confocal microscopy (Leica, Germany). Tail moment was analyzed using Image J.

### Purification of recombinant proteins

WT and mutant GST-PGK1, WT and mutant His-PGK1, WT and mutant His-MORC2, WT and mutant GST-MORC2, and His-CK2 proteins were expressed in bacteria and purified as described previously [29]. Briefly, corresponding constructs were expressed in the BL21 bacterial strain. The bacteria were cultured at 37 °C until the OD reached 0.6. Protein expression was induced with 0.5 mM IPTG overnight. For purification of His-tagged proteins, cleared lysates were loaded onto a Ni-NTA column, washed with five column volumes of 20 mM imidazole and subsequently eluted with 250 mM imidazole. For purification of GST-tagged proteins, cleared lysates were loaded onto a GSTrap HP column, washed with five column volumes of PBS, and subsequently eluted with 10 mM reduced glutathione. The proteins were desalted in 10-kDa spin columns via washing twice with ice-cold PBS.

### In vitro kinase assay

Kinase assays were performed as described previously [30]. Briefly, bacterially purified recombinant MORC2 (500 ng) was added to kinase assay buffer (CST) kinase assay buffer (25 mM Tris-HCl [pH 7.5], 5 mM β-glycerophosphate, 2 mM dithiothreitol (DTT), 10 mM MgCl_2_ and 0.1 mM Na_3_VO_4_) and incubated with PGK1 (100 ng) and CK2 (100 ng) and 50 μM ATP-γ-S at 30 °C for 45 min. The samples were alkylated with 50 mM PNBM/5% DMSO, incubated for 1 h at 25 °C, and then subjected to SDS-PAGE and immunoblotting. Proteins phosphorylated by ATP-γ-S were detected using an anti-thiophosphate ester antibody (Abcam).

### ATPase assays

ATPase assays were performed using -γ-^32^P-labeled ATP as described previously [31]. Reaction mixtures (20 μL) contained 30 mM Tris-Cl (pH 7.4), 0.1 M NaCl, 8 mM MgCl_2_, 1 mM DTT, 0.5 μCi [γ-^32^P] ATP, 0.5 μM MORC2 and 100 ng of plasmid DNA. Samples were incubated at 30 °C for 20 min, reactions were quenched by the addition of 2 × formamide loading buffer (0.1% bromophenol blue, 90% deionized formamide and xylene cyanol), and 4 μL was resolved on a 12% polyacrylamide gel (19 : 1 acrylamide to bisacrylamide) containing 7 M urea for 1.5 h at 100 V. Wet gels were autoradiographed.

### Phosphatase assay

ATPase activity of purified MORC2 proteins were performed by estimating inorganic phosphate liberated from ATP during enzymatic reaction. In brief, 1 µg of each protein were incubated with100 ng of plasmid DNA, 150 µM of ATP in buffer containing 20 mM HEPES (pH 7.4), 500 mM NaCl, 5 mM KCl and 10 mM MgCl_2_ for 1 h at room temperature. Reactions were quenched by the addition of EDTA. Amount of inorganic phosphate was measured using a Malachite Green Assay Kit (Sigma-Aldrich).

### Immunohistochemical (IHC) staining and scoring

All PDAC specimens were paraffin embedded and collected from the Beijing Luhe Hospital. A total of 60 paired PDAC tumor and adjacent normal tissue specimens were obtained via surgical resection or needle biopsy. None of these patients had received prior radiotherapy or chemotherapy before tissue sampling. Among these 60 PDAC cases: 51 patients with stage I/II disease underwent radical resection followed by adjuvant chemotherapy or radiotherapy, 9 patients received palliative chemotherapy alone. Tumor specimens from this cohort were subjected to IHC analysis of PGK1 expression. No significant difference in post-diagnosis treatment strategies was observed between PGK1 low-expression and high-expression subgroups. Additionally, an independent cohort of 37 treatment-naive stage III PDAC patients received neoadjuvant chemoradiotherapy followed by radical surgery. Tumor specimens from this cohort were collected post-radiotherapy and subjected to IHC analysis of PGK1 pS256 and MORC2 pS711 expression. All clinicopathological diagnoses were independently verified by two board-certified pathologists according to the AJCC Cancer Staging Manual (8th Edition, 2017). Detailed information on the patient characteristics is presented in Table S3.

Sections of paraffin-embedded tissue were stained with the indicated antibody. IHC staining was performed using a VECTASTAIN ABC kit according to the manufacturer’s instructions. The IHC scores were evaluated by two independent authors blinded to the patients’ clinicopathological data. We quantitatively scored the tissue sections according to the percentage of positive cells (1, 0–25%; 2, 26–50%; 3, 51–75%; and 4, >75%) and staining intensity (0, no staining; 1, weak staining; 2, moderate staining; and 3, strong staining). These numbers were then multiplied, resulting in a score of 0–12 as described previously [32]. The specimens with scores ≥4 were classified as high-expression, while those with scores < 4 were classified as low-expression. Scores were compared with the overall survival, which was defined as the time from the date of surgery to death or the last known date of follow-up.

### Proliferation assays

Cellular metabolic activity was evaluated using a CCK-8 assay. Cells (5 × 10³/well) were seeded in 96-well plates. Following doxorubicin treatment for indicated durations, 10 μL of CCK-8 reagent was added to each well, and plates were incubated at 37 °C for 30 min. Viability was quantified by measuring absorbance at 450 nm using a microplate reader.

### Cell cycle analysis

Cell cycle phase quantification was performed with a commercial detection kit (BD Biosciences, Cat#550825). Cells underwent fixation in ice-cold 70% ethanol (4 °C, overnight). Fixed cells were then pelleted and resuspended in 500 μL staining buffer. After 15-min incubation at room temperature protected from light, samples were subjected to flow cytometric analysis (BD Biosciences). Cell cycle phase distribution (G₀/G₁, S, G₂/M) was determined using Flowjo software (Version 10.8.1, BD Biosciences, USA).

### Extracellular acidification rate (ECAR) and oxygen consumption rate (OCR) assay

ECAR and OCR were quantified using an XF96 Extracellular Flux Analyzer (Seahorse Bioscience). Cells were seeded in XF96 microplates (1.0 × 10⁴ cells/well) and cultured overnight. One hour prior to measurement, growth medium was replaced with XF assay medium. XF Glycolysis Stress Test Kit was used to measure the glycolytic capacity. Glucose, oligomycin and 2-deoxy glucose (2-DG) were diluted into XF media and loaded into the cartridge to achieve final concentrations of 10, 1, and 50 mM, respectively. ECAR measurements followed manufacturer protocols. Mitochondrial respiratory function was assessed with the XF Cell Mito Stress Test Kit. Oligomycin, FCCP, antimycin and rotenone were diluted into XF media and loaded into the cartridge to achieve final concentrations of 1, 1, 5, and 1 μM, respectively. OCR values were recorded according to standardized procedures. Protein concentrations were determined by BCA protein assay kit and the real time ATP rate, OCR and ECAR were normalized by the total protein.

### Micrococcal nuclease assay

Approximately 2 × 10⁶ PDAC cells were seeded in 6-cm dishes. Cells were either untreated or treated with 4 Gy IR and harvested 1 h post-irradiation. Cells were scraped off and centrifuged at 1000 rpm for 3 min. The pellet was resuspended in hypotonic lysis buffer (10 mM Tris-HCl pH 8.0, 10 mM MgCl₂, 1 mM DTT, 0.5% NP-40) and incubated on ice for 8 min. After centrifugation (1000 rpm, 3 min), the supernatant was discarded. The remaining pellet (nuclei) was resuspended in 200 μL Digestion Buffer (15 mM Tris-HCl pH 7.4, 60 mM KCl, 15 mM NaCl, 0.25 M sucrose, 1 mM CaCl₂, 0.5 mM DTT). 4U of Micrococcal Nuclease (MNase, Sigma, Cat#N3755) was added to the nuclear suspension and mixed thoroughly. Digestion proceeded for 5 min at 37 °C. Genomic DNA was then extracted using phenol-chloroform extraction. The purified DNA was separated by electrophoresis in 1.2% agarose gel. Band intensities at 200 bp, 400 bp, and 600 bp were quantified using ImageJ software.

### Chromatin isolation

Chromatin extraction was performed as described previously [33]. Briefly, 4 × 10^6^ cells treated with or without IR were washed with PBS and scraped from the dishes in the presence of lysis buffer A (10 mM HEPES, pH7.9, 1.5 mM MgCl_2_, 10 mM KCl 0.34 M sucrose, 10% glycerol, 1 mM DTT, 1 × protease inhibitor cocktail and 1 × phosphatase inhibitor cocktail). Triton X-100 was added to a final concentration of 0.05% followed by incubation on ice for 5 min. Nuclei were collected by centrifugation at 14,000 rpm for 5 min. Nuclei were washed once with solution A and lysed with solution B (0.2 mM EGTA, 3 mM EDTA, 1 mM DTT, 1 × protease inhibitor cocktail, and 1 × phosphatase inhibitor cocktail) on ice for 10 min. The insoluble chromatin fraction was collected by centrifugation at 2000 rpm for 4 min at 4 °C, washed once in solution B, and centrifuged again at 14,000 rpm for 1 min. The final chromatin pellet was resuspended in Laemmli buffer and sonicated for 15 s.

### Salt solubilization assays

Briefly, nuclei were isolated from 2 × 10^6^ cells using hypotonic lysis and were incubated in non-denaturing extraction buffers (20 mM Tris-Cl, pH 7.6, 5% glycerol) supplemented with 100 to 500 mM NaCl in the presence of protease inhibitor cocktail and phosphatase inhibitor cocktail for 5 min. Nuclei were collected by centrifugation at 700 *g* for 20 min. Salt soluble fractions were obtained by centrifugation, resolved by SDS-PAGE and analyzed by immunoblotting.

### Materials

All antibodies and reagents are listed in Table S4.

### Quantification and statistical analysis

All non-animal experiments were independently repeated at least three times, with representative results shown. For animal studies and patient-derived tissue experiments, sample sizes were chosen empirically based on our previous experience in the calculation of experimental variability. All statistics were performed and generated using SPSS software. Statistical differences were determined using either Student’s *t* test or one-way analysis of variance (ANOVA), as indicated in the corresponding figure legends. The survival analysis was plotted by the Kaplan–Meier method and were compared by the log-rank test. Differences were considered statistically significant when the *p* value was less than 0.05. All experiments were performed in triplicate unless otherwise noted. Data are presented as mean ± SD for continuous variables and as frequencies and proportions for categorical variables. ∗∗∗*p* ≤ 0.001, ∗∗*p* ≤ 0.01, ∗*p* ≤ 0.05.

## Results

### PGK1 is highly expressed in PDAC specimens and indicates poorer prognosis

The expression levels of PGK1 in PDAC tissues and normal pancreatic tissues were compared using the GEPIA database. PGK1 was found to be significantly upregulated in PDAC tissues compared to normal tissues (Fig. S2A). This observation prompted us to investigate the clinical significance of PGK1 expression in PDAC. Analysis of disease-free survival (DFS) and overall survival (OS) data from the GEPIA database revealed that high PGK1 expression was associated with worse DFS and OS in PDAC patients (Fig. S2B and S2C). These findings suggested that PGK1 might serve as a prognostic biomarker for PDAC.

**Fig. 1:**
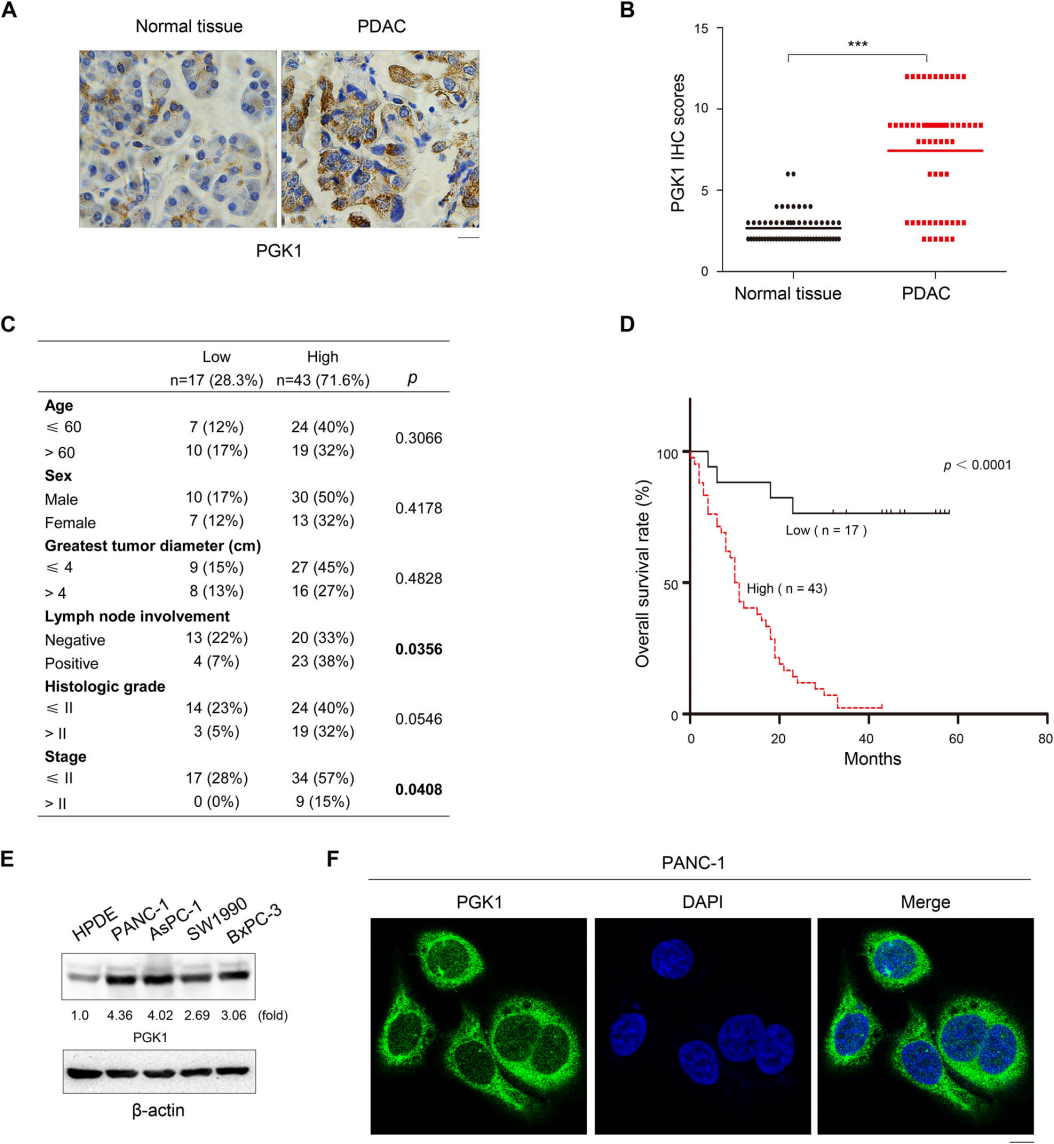
PGK1 is highly expressed in PDAC specimens and indicates poorer prognosis. **A** Expression of PGK1 in 60 samples of human PDAC tissues and matched adjacent normal tissues by IHC staining with an anti-PGK1 antibody. Representative images are shown. Scale bars, 20 μm. **B** Comparative analysis of PGK1 expression between PDAC tissues and matched adjacent normal tissues. ***p < 0.001. **C** Correlations between PGK1 expression levels and PDAC clinicopathological parameters. **D** Kaplan–Meier plots and *p*-values of the log-rank test for comparing survivals of PDAC patients with high and low expression of PGK1. *p* < 0.0001. **E** Lysates of the indicated cells were prepared. Immunoblot analyses were performed with the indicated antibodies. **F** IF analyses of PANC-1 cells were performed with the indicated antibodies. Nuclei were stained with DAPI. Scale bars, 10 μm.

To validate these findings, we performed IHC staining on 60 pairs of primary PDAC tumor specimens and their matched normal tissues using an anti-PGK1 antibody. The results showed that PGK1 expression was significantly higher in PDAC tissues than in adjacent normal tissues (Fig. 1A, B). The TNM staging system (T: primary tumor extent, N: regional lymph node involvement, M: distant metastasis status) encapsulates the tumor biology, characterizes its aggressive behavior, and informs patient prognosis. PGK1 expression levels were positively correlated with lymph node involvement and advanced TNM stage in PDAC patients (*p* < 0.05) (Fig. 1C). Kaplan–Meier analysis further confirmed that high PGK1 expression was significantly associated with decreased OS durations in PDAC patients (Fig. 1D).

Consistent with the clinical data, we observed higher PGK1 expression levels in several human PDAC cell lines (PANC-1, AsPC-1, SW1990, and BxPC-3) compared to normal human pancreatic duct epithelial (HPDE) cells (Fig. 1E). IF analyses demonstrated that PGK1 was localized in the nucleus of PANC-1 cells (Fig. 1F). Collectively, these results indicate that PGK1 is highly expressed in PDAC tissues and cell lines, and its elevated expression is associated with poorer prognosis in PDAC patients.

### PGK1 facilities radioresistance of PDAC by inhibiting DNA damage response

**Fig. 2:**
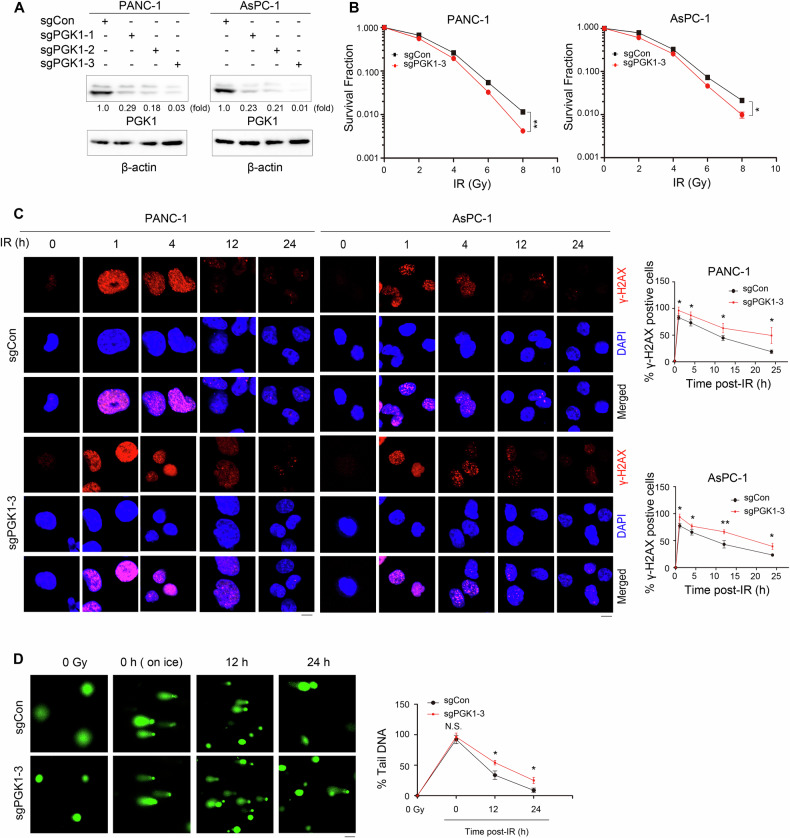
PGK1 facilities radioresistance of PDAC by inhibiting DNA damage response. **A** PGK1 was depleted in the indicated cells by expressing PGK1 sgRNA. Immunoblot analyses were performed with the indicated antibodies. **B** The PANC-1 and AsPC-1 with or without expressing PGK1 sgRNA-3 were exposed to or not to IR. Clonogenic survival assays were performed in indicated cells. Data are presented as the means ± SD from three independent experiments (*n* = 3). *p < 0.05 or **p < 0.01 using unpaired Student’s *t* test. **C** PGK1 was depleted in the indicated cells by expressing PGK1 sgRNA-3. The indicated PANC-1 and AsPC-1 cells were exposed to or not to IR (4 Gy). The indicated cells were harvested at different time points for IF analyses. Data are presented as mean ± SD from 3 independent experiments (*n* = 3). *p < 0.05 or **p < 0.01 using unpaired Student’s *t* test. Scale bars, 10 μm. **D** The indicated PANC-1 cells were exposed to or not to IR (4 Gy) and then subjected to alkaline comet assay at indicated time points. Tail moment was quantified and graphed for each group. *p < 0.05 using unpaired Student’s *t* test. Scale bar, 50 µm.

PGK1 has been reported to mediate cisplatin chemoresistance through DNA repair mechanisms [34]. To investigate whether PGK1 similarly contributes to radioresistance, we assessed the survival of PDAC cells following irradiation using clonogenic formation assays. Our results demonstrated that IR significantly diminished the colony-forming capacity of PANC-1 and AsPC-1 cells in a dose-dependent manner. Notably, this effect was partially aggravated by PGK1 depletion (Fig. 2A, B). Conversely, overexpression of PGK1 in these cells (Fig. S3A) rendered them less sensitive to IR (Fig. S3B). Collectively, these findings indicate that PGK1 plays a role in conferring radioresistance in PDAC cells. Additionally, IR exposure did not alter PGK1 expression levels at either the mRNA or protein level (Fig. S3C and S3D). We further examined the impact of PGK1 depletion on DNA damage response by analyzing γ-H2AX foci formation, a well-established marker for DNA double-strand breaks (DSBs). IR exposure induced a peak of γ-H2AX foci within 1 h in PANC-1 and AsPC-1 cells, which persisted for up to 24 h. PGK1 depletion significantly slowed the decline in γH2AX foci levels induced by IR, while PGK1 overexpression accelerated this decline. (Fig. 2C, Fig. S3E). In addition to IR, Doxorubicin (DOX) is a widely-used anti-cancer drug that induces DNA damage. We similarly found that PGK1 depletion rendered PANC-1 and AsPC-1 cells more sensitive to DOX (Fig. S3F). Notably, treatment with DOX led to a rapid increase in γ-H2AX foci in PANC-1 and AsPC-1 cells, and this increase was further potentiated by PGK1 depletion (Fig. S3G). The increased sensitivity of PDAC cells to DOX upon PGK1 knockdown further supports the role of PGK1 in regulating the DNA damage response, rather than implying clinical treatment relevance.

To assess whether PGK1 depletion contributed to increased DNA damage induction or delayed repair, we performed the alkaline comet assay, a method that quantitatively evaluates single- and double-strand DNA breaks [35]. When performed at warmer temperatures and with longer incubation times post-IR exposure, this assay reflects both the induction and repair of DNA damage. Conversely, when performed on ice to arrest DNA repair, it measures only the induction of DNA damage. Our findings revealed that PGK1 depletion did not affect the amount of DNA damage induced when cells were irradiated on ice and harvested immediately (Fig. 2D). However, PGK1 depletion significantly increased the amount of DNA damage present after repair was allowed to proceed at 37 °C for 12 and 24 h in PANC-1 cells (Fig. 2D). These results indicate that PGK1 enhances the repair of IR-induced DNA damage in PDAC cells, thereby contributing to radioresistance.

### IR exposure results in CK2-dependent phosphorylation of PGK1 at Ser256 and subsequent PGK1/MORC2 complex formation

**Fig. 3:**
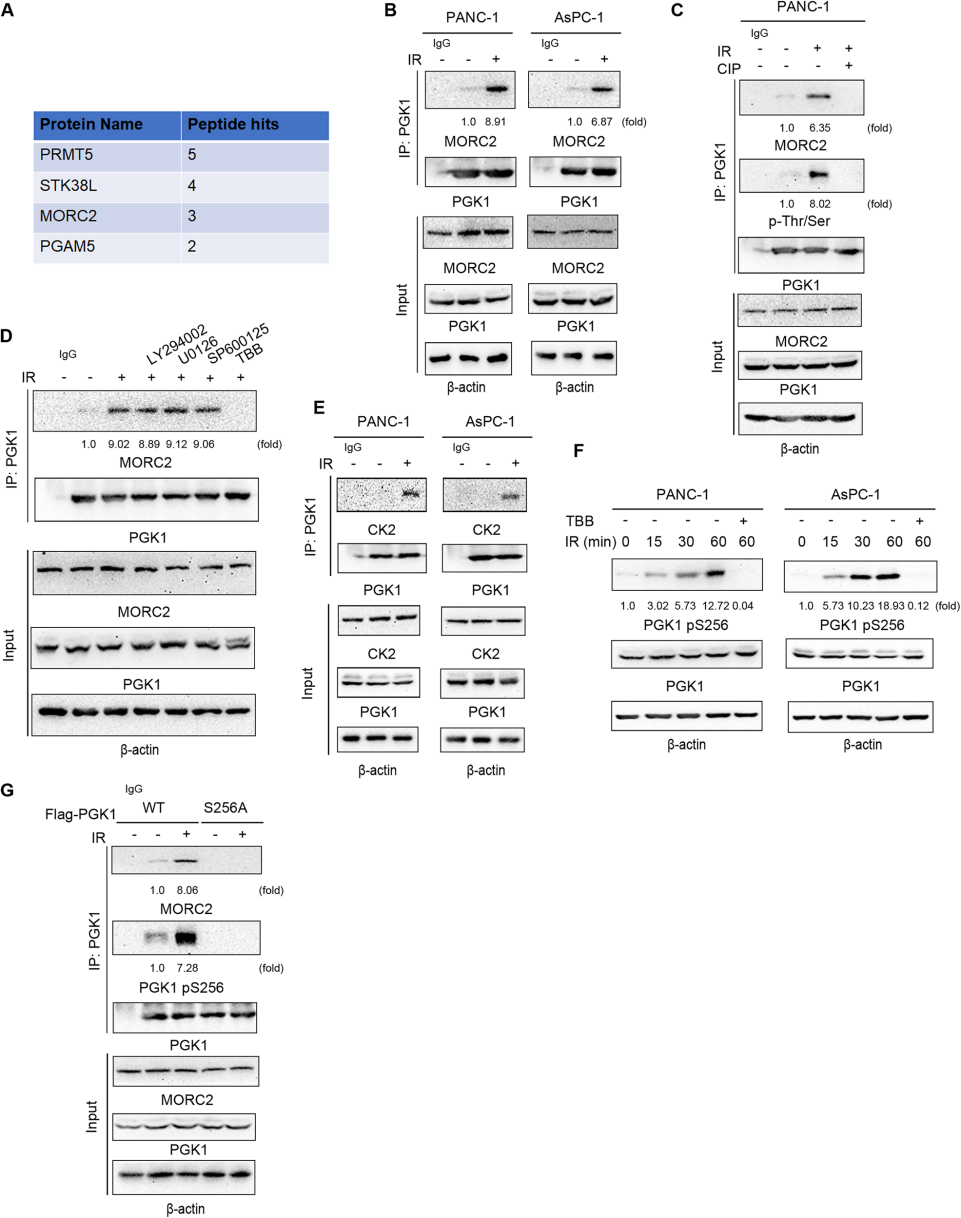
IR exposure results in CK2-dependent phosphorylation of PGK1 at Ser256 and subsequent PGK1/MORC2 complex formation. **A** PANC-1 cells were collected 30 min after exposure to IR. PGK1 in the PANC-1 cells was immunoprecipitated with an anti-PGK1 antibody. The immunoprecipitates were analyzed by mass spectrometry. PGK1-interacting proteins were identified by mass spectrometry. **B** The PANC-1 and AsPC-1 cells were collected 6 h after exposure to IR (4 Gy). Immunoprecipitation with an anti-PGK1 antibody was performed. **C** PANC-1 cells were collected 6 h after exposure to IR (4 Gy). The immunoprecipitates were treated with or without CIP (10 units) and analysed by immunoblotting. **D** PANC-1 cells were pretreated with or without LY294002 (30 μM), U0126 (20 µM), SP600125 (25 μM), TBB (10 μM) for 1 h before exposure to IR (4 Gy) for 6 h. Immunoprecipitation and immunoblot analyses were performed with the indicated antibodies. **E** The PANC-1 and AsPC-1 cells were collected 6 h after exposure to IR (4 Gy). Immunoprecipitation with an anti-PGK1 antibody was performed. **F** The PANC-1 and AsPC-1 cells pretreated with or without TBB (10 μM) for 1 h were exposure to IR (4 Gy) for the indicated periods. Total cell lysates were prepared. Immunoblotting with the indicated antibodies was performed. **G** PANC-1 cells expressing WT Flag-rPGK1 or Flag-rPGK1 S256A were exposure to or not to IR (4 Gy) for 6 h. Immunoprecipitation and immunoblot analyses were performed with the indicated antibodies.

To unravel the mechanisms behind PGK1-mediated radioresistance, we employed mass spectrometry analyses on PGK1 immunoprecipitates derived from irradiated PDAC cells. Intriguingly, these analyses highlighted that MORC2, an ATPase implicated in chromatin remodeling and DNA damage repair, interacted with PGK1 following IR exposure (Fig. 3A). This interaction was subsequently confirmed through co-immunoprecipitation assays in PANC-1 and AsPC-1 cells, revealing that IR significantly enhanced the binding between endogenous PGK1 and MORC2 (Fig. 3B). Notably, treatment with calf intestinal phosphatase (CIP) effectively abolished this interaction, suggesting the interaction is phosphorylation-dependent (Fig. 3C).

Investigating the kinase responsible for this phosphorylation event, we treated PDAC cells with a panel of inhibitors targeting IR stress-responsive kinases. Among these, only the CK2 inhibitor effectively blocked the IR-induced interaction between PGK1 and MORC2 (Fig. 3D and S4A). As previously documented, CK2 phosphorylates nuclear PGK1 at serine 256 (S256), facilitating PGK1’s interaction with CDC7 and thereby promoting DNA replication [16]. Consistent with these findings, our co-immunoprecipitation assays demonstrated that IR exposure induced the interaction between PGK1 and CK2 in PANC-1 and AsPC-1 cells (Fig. 3E). Additionally, IR exposure triggered phosphorylation of PGK1 at S256, an event that was effectively inhibited by pre-treatment with a CK2 inhibitor (Fig. 3F). Immunoprecipitation analysis further revealed that wild-type (WT) PGK1, but not the S256A mutant, bound to MORC2 in response to IR exposure in PANC-1 cells (Fig. 3G). Collectively, these data indicate that CK2-mediated phosphorylation of PGK1 at S256 promotes the formation of the PGK1/MORC2 complex.

### PGK1 phosphorylates MORC2 at S711

**Fig. 4:**
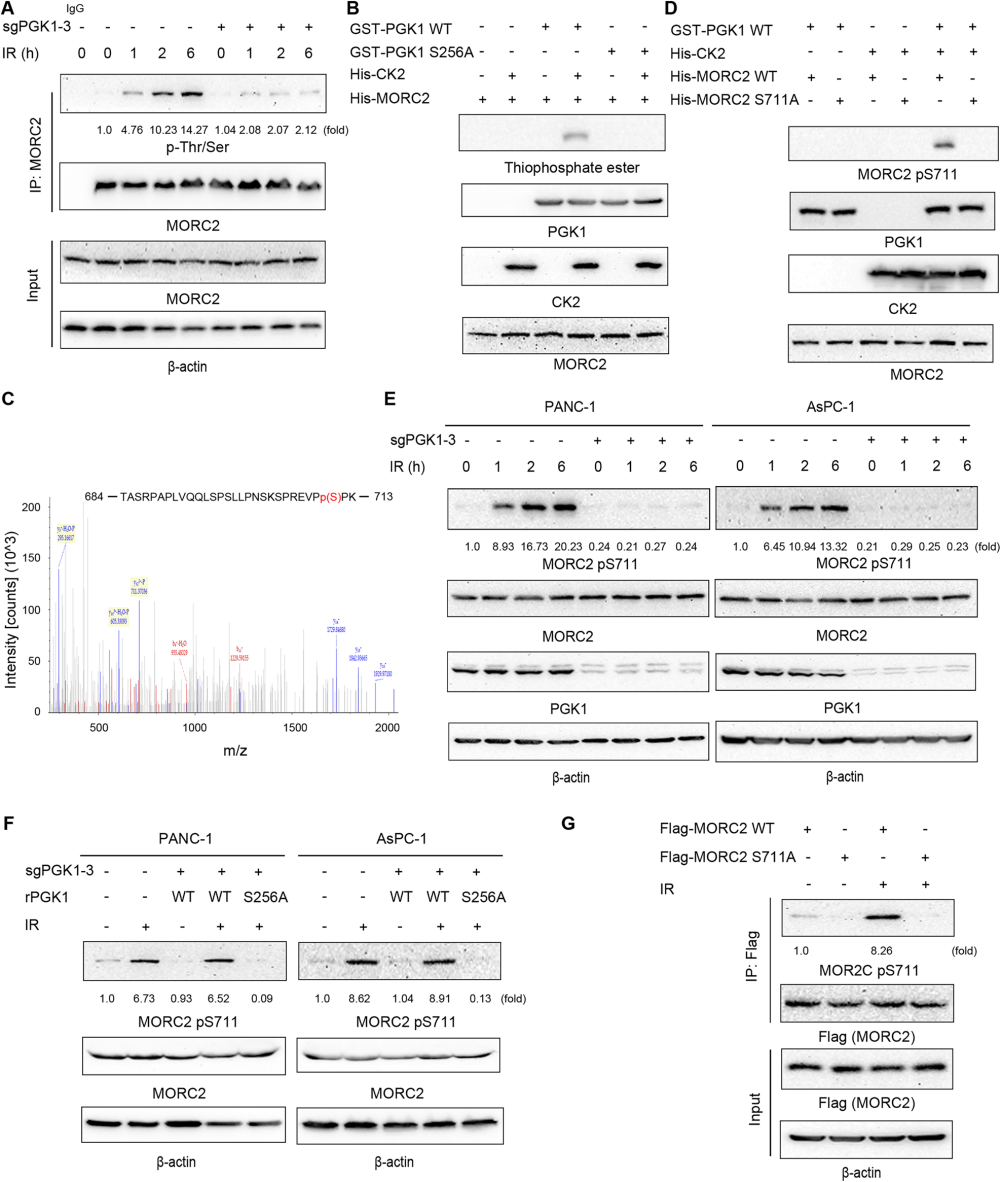
PGK1 phosphorylates MORC2 at S711. **A** PANC-1 cells with or without expressing PGK1 sgRNA-3 were exposure to or not to IR (4 Gy) for 6 h. Immunoprecipitation and immunoblot analyses were performed with the indicated antibodies. **B**, **C** In vitro phosphorylation analyses were performed by mixing purified His-CK2 with the indicated purified His-MORC2 and GST-PGK1 or GST-PGK1 S256A protein in the presence of ATP-γ-S. Immunoblot analyses were performed with the indicated antibodies (**B**). GST-MORC2 protein was immunoprecipitated with an anti-GST antibody and analyzed by mass spectrometry. The results of mass spectrometry showed that MORC2 S711 was phosphorylated (**C**). **D** In vitro phosphorylation analyses were performed by mixing purified His-CK2 with the indicated purified GST-PGK1 and His-MORC2 WT or His-MORC2 S711A protein in the presence of ATP-γ-S. Immunoblot analyses were performed with the indicated antibodies. **E** The PANC-1 and AsPC-1 cells with or without expressing PGK1 sgRNA were exposure to or not to IR (4 Gy) for the indicated periods. Immunoblot analyses were performed with the indicated antibodies. **F** The PANC-1 and AsPC-1 cells with endogenous PGK1 depletion and reconstituted expression of WT rPGK1 or rPGK1 S711A were exposure to or not to IR (4 Gy) for 6 h. Immunoblot analyses were performed with the indicated antibodies. **G** PANC-1 cells expressing WT Flag-MORC2 or Flag-MORC2 S711A were exposure to or not to IR (4 Gy) for 6 h. Immunoprecipitation and immunoblot analyses were performed with the indicated antibodies.

Given that the PGK1-catalyzed conversion of 1,3-bisphosphoglycerate (1,3-BPG) to 3-phosphoglycerate (3-PG) and ATP is reversible, with PGK1 utilizing ATP as a phosphate donor [14, 15], we hypothesized that PGK1 might phosphorylate MORC2. Indeed, we observed that IR induced MORC2 Thr/Ser phosphorylation in PANC-1 cells, an effect that was significantly attenuated by PGK1 depletion (Fig. 4A). To confirm this, we conducted an in vitro phosphorylation assay, which revealed that purified WT GST-PGK1, but not GST-PGK1 S256A, phosphorylated WT His-MORC2 (Fig. 4B). Furthermore, PGK1 specifically phosphorylated MORC2 and not the GST-tag protein (Fig. S5A). Mass spectrometry analysis pinpointed serine 711 (S711) as the specific phosphorylation site on MORC2 (Fig. 4C). Consistent with these findings, only purified WT MORC2, and not the MORC2 S711A mutant, was phosphorylated by PGK1. This phosphorylation was further validated using a specific anti-MORC2 pS711 antibody (Fig. 4D and S5B). PGK1 WT, but not the PGK1 T378P kinase-dead mutant [15], phosphorylated MORC2 S711 (Fig. S5C). Moreover, PGK1 catalyzes the phosphorylation of MORC2 S711 in a concentration-dependent manner (Fig. S5D). Additionally, IR exposure rapidly induced MORC2 S711 phosphorylation (Fig. 4E), an effect that was abolished by PGK1 depletion and restored by reconstituted expression of WT Flag-rPGK1, but not by the Flag-rPGK1 S256A mutant in PANC-1 and AsPC-1 cells (Fig. 4F and S5E). Similarly, IR induced S711 phosphorylation of WT MORC2, but not the MORC2 S711A mutant, in PANC-1 cells (Fig. 4G). Collectively, these results demonstrate that CK2-dependent PGK1 S256 phosphorylation facilitates PGK1 binding to MORC2 and subsequent phosphorylation of MORC2 at S711.

### PGK1-phosphorylated MORC2 S711 promotes its DNA-dependent ATPase activity

**Fig. 5:**
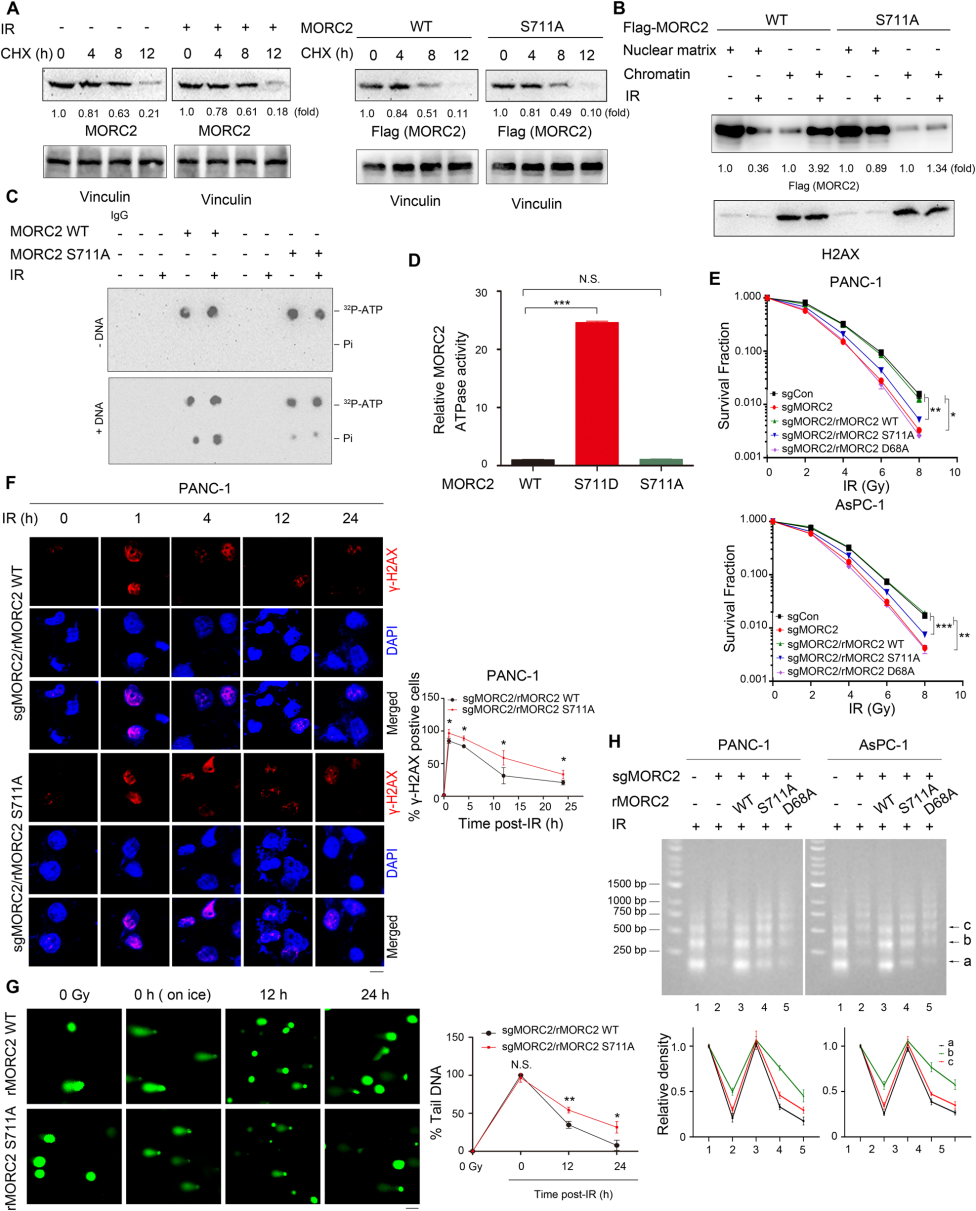
PGK1-phosphorylated MORC2 S711 promotes its DNA-Dependent ATPase activity. **A** PANC-1 cells pretreated with or without CHX (100 μg/ml) for 1 h were exposure to IR (4 Gy) for the indicated periods. Immunoblotting with the indicated antibodies was performed (left panel). PANC-1cells expressing WT Flag-rMORC2 or Flag-rMORC2 S711A were treated with or without 100 μg/ml of CHX for the indicated times and analyzed by immunoblotting (right panel). **B** Subcellular fractions were prepared from PANC-1 cells expressing WT Flag-MORC2 or Flag-MORC2 S711A after 1 h of IR (4 Gy) exposure and immunoblotted with the indicated antibodies. **C** PANC-1 cells expressing WT Flag-MORC2 or Flag-MORC2 S711A were exposure to IR (4 Gy). After 1 hr of IR treatment, nuclear extracts were immunoprecipitated with Flag-tagged beads and subjected to ATPase assays using double-stranded plasmid DNA. **D** Purified bacteria-expressed WT His-MORC2, MORC2 S711D or MORC2 S711A proteins were incubated with DNA and the released phosphate was measured. Data is presented as mean ± SD from 3 independent experiments (n = 3). N.S., not significant for the indicated comparison or ***p < 0.001 using one-way ANOVA, followed by Bonferroni’s post hoc test. **E** The PANC-1 and AsPC-1 cells with endogenous MORC2 depletion and reconstituted expression of Flag-rMORC2, Flag-rMORC2 S711A or Flag-rMORC2 D68A were exposure to or not to IR. Clonogenic survival assays were performed in the indicated cells. Data are presented as the means ± SD from three independent experiments (*n* = 3). *p < 0.05, **p < 0.01 or ***p < 0.001 using one-way ANOVA, followed by Bonferroni’s post hoc test. **F**, **G** The PANC-1 cells with endogenous MORC2 depletion and reconstituted expression of Flag-rMORC2 or Flag-rMORC2 S711A were exposure to or not to IR (4 Gy). The indicated cells were harvested at different time points for IF analyses (**F**) and alkaline comet assay (**G**). Data are presented as mean ± SD from 3 independent experiments s (*n* = 3) (**F**). N.S., not significant for the indicated comparison, *p < 0.05 or **p < 0.01using unpaired Student’s *t* test. Scale bars, 10 μm (**F**), 50 μm (**G**). **H** PANC-1 and AsPC-1 cells transduced with control sgRNA or MORC2 sgRNA vectors, followed by reconstitution with WT rMORC2, rMORC2 S711A or rMORC2 D68A, were exposure to IR (4 Gy). After 1 h of IR treatment, nuclear extracts were incubated with MNase for 5 min, and DNA was visualized by ethidium bromide staining. The band (a, 200 bp, b, 400 bp, and c, 600 bp) densities were quantified using ImageJ software, and the results were normalized to the signal for a1, b1, and c1, respectively. Data are presented as the means ± SD from three independent experiments (*n* = 3).

Protein phosphorylation, a prevalent post-translational modification (PTM) in eukaryotes, is pivotal for regulating enzyme activities by modulating their stability and localization [36]. To elucidate whether PGK1-dependent phosphorylation of MORC2 at S711 impacts MORC2 stability, we conducted cycloheximide (CHX) chase assays. Our results demonstrated that IR exposure did not alter the half-life of MORC2, and the MORC2 S711A mutant exhibited a comparable half-life to its WT counterpart in PANC-1 cells following IR exposure (Fig. 5A). Additionally, we assessed the subcellular localization of WT MORC2 and MORC2 S711A in PANC-1 cells. The result showed that following IR WT MORC2 protein became more strongly associated with chromatin as compared with the MORC2 S711A, suggesting that PGK1-phosphorylated MORC2 S711 regulates its association with chromatin following DNA damage (Fig. 5B). As MORC2 associates with chromatin and possesses an DNA-dependent ATPase activity following DNA damage, we examined whether PGK1-dependent phosphorylation of MORC2 at S711 directly influenced its enzymatic activities. The ATPase activity of WT MORC2 was significantly stimulated by IR treatment in the presence of DNA, whereas the MORC2 S711A mutant showed no such increase (Fig. 5C). To further validate this, we depleted endogenous PGK1 and reconstituted expression with either WT V5-rPGK1 or the V5-rPGK1 S256A mutant in PANC-1 cells. The ATPase activity of MORC2 was compromised in the presence of DNA when PGK1 was depleted and reconstituted with the S256A mutant, but not with WT V5-rPGK1 (Fig. S6A). Consistent with these findings, purified MORC2 S711D (a phosphorylation-mimicking mutant) showed enhanced activity comparable to the WT and MORC2 S711A (Fig. 5D).

To determine whether PGK1-dependent MORC2 S711 phosphorylation contributes to radioresistance, we examined the survival of MORC2-depleted PANC-1 and AsPC-1 cells reconstituted with either WT rMORC2, the phosphor-defective rMORC2 S711A mutant, or the ATPase-dead rMORC2 D68A mutant (where the catalytic residue D68 is essential for ATP hydrolysis and binding) [22] (Fig. S6B) following exposure to IR using clonogenic formation assays. The results demonstrated that IR significantly reduced colony formation in both cell lines. MORC2 depletion partially aggravated this effect, indicating its role in promoting radioresistance. Reconstitution with WT rMORC2, but not the S711A or D68A mutants, restored radioresistance in depleted cells (Fig. 5E). These findings collectively indicate that MORC2 regulates IR sensitivity through its DNA-dependent ATPase activity, which requires phosphorylation at S711. Furthermore, depletion of endogenous MORC2 and reconstitution with the S711A mutant, but not WT rMORC2, impaired the ability of PDAC cells to repair IR-induced DSBs, as evidenced by increased γ-H2AX foci (Fig. 5F and S6C) and alkaline comet assay results (Fig. 5G). As MORC2 exhibits intrinsic DNA-dependent ATPase activity following DNA damage, we next examined whether PGK1-catalyzed phosphorylation of MORC2 at S711 alters chromatin structure by analyzing chromatin sensitivity to micrococcal nuclease (MNase). Increased MNase sensitivity is generally interpreted as reflecting relaxed chromatin conformation, which undergoes accelerated digestion by MNase and is frequently associated with DNA repair processes [37]. We found that MORC2 knockout resulted in a moderate reduction in MNase accessibility in chromatin following IR treatment. Reconstitution with MORC2 WT in endogenous MORC2-depleted cells restored MNase sensitivity. In contrast, chromatin from cells reconstituted with either the phosphor-defective MORC2 S711A mutant or the ATPase-dead MORC2 D68A mutant failed to show significant restoration of MNase sensitivity (Fig. 5H). Furthermore, salt solubilization assays demonstrated that reconstitution with the S711A MORC2 mutant markedly attenuated DNA damage-induced dissociation of core histones from chromatin compared to WT MORC2 reconstitution in both PANC-1 and AsPC-1 cells with endogenous MORC2 knockout (Fig. S6D). Collectively, these findings suggest that MORC2 functions as a DNA-dependent ATPase whose activity requires PGK1-mediated phosphorylation at S711 following DNA damage.

IR induces phosphorylation of PGK1 at S256, thereby promoting the PGK1-MORC2 interaction and catalyzing phosphorylation of MORC2 at S711, which enhances the ATPase activity of MORC2. To investigate whether PGK1-mediated regulation of MORC2 ATPase activity depends on PGK1’s metabolic enzyme function, we reconstituted endogenously PGK1-depleted PANC-1 and AsPC-1 cells with either WT PGK1 or the S256 A mutant, and quantified changes in ECAR/OCR metabolic flux, cell cycle distribution, and proliferation rates. Consistent with reports in the literature[16], our results shown PGK1 depletion caused a significant reduction in the ECAR in PANC-1 and AsPC-1 cells, indicating decreased glycolytic activity, while having no significant effect on OCR (Fig. S7A). Additionally, PGK1 depletion induced cell cycle arrest at in G0/G1 phase and inhibited cell proliferation (Fig. S7B and S7C). Reconstitution with either WT rPGK1 or the rPGK1 S256 A mutant restored the alterations in ECAR, cell cycle, and proliferation caused by PGK1 depletion, indicating that the S256A mutation does not impair PGK1’s metabolic function. Furthermore, PGK1 depletion sensitized PANC-1 and AsPC-1 cells to IR. Reconstitution of WT rPGK1 expression completely reversed this radiosensitization, whereas reconstitution with the S256A mutant failed to rescue the phenotype (Fig. S7D). The MNase Assay showed that PGK1 knockout moderately reduced MNase accessibility in chromatin after IR. Reconstitution with WT PGK1 restored MNase sensitivity, but cells expressing the S256A mutant showed no significant restoration (Fig. S7E). These results demonstrate that PGK1 S256 phosphorylation does not affect PGK1’s metabolic enzyme activity, but can modulate the DNA-dependent ATPase activity of MORC2.

### PGK1-mediated MORC2 S711 phosphorylation promotes radioresistance of PDAC

**Fig. 6:**
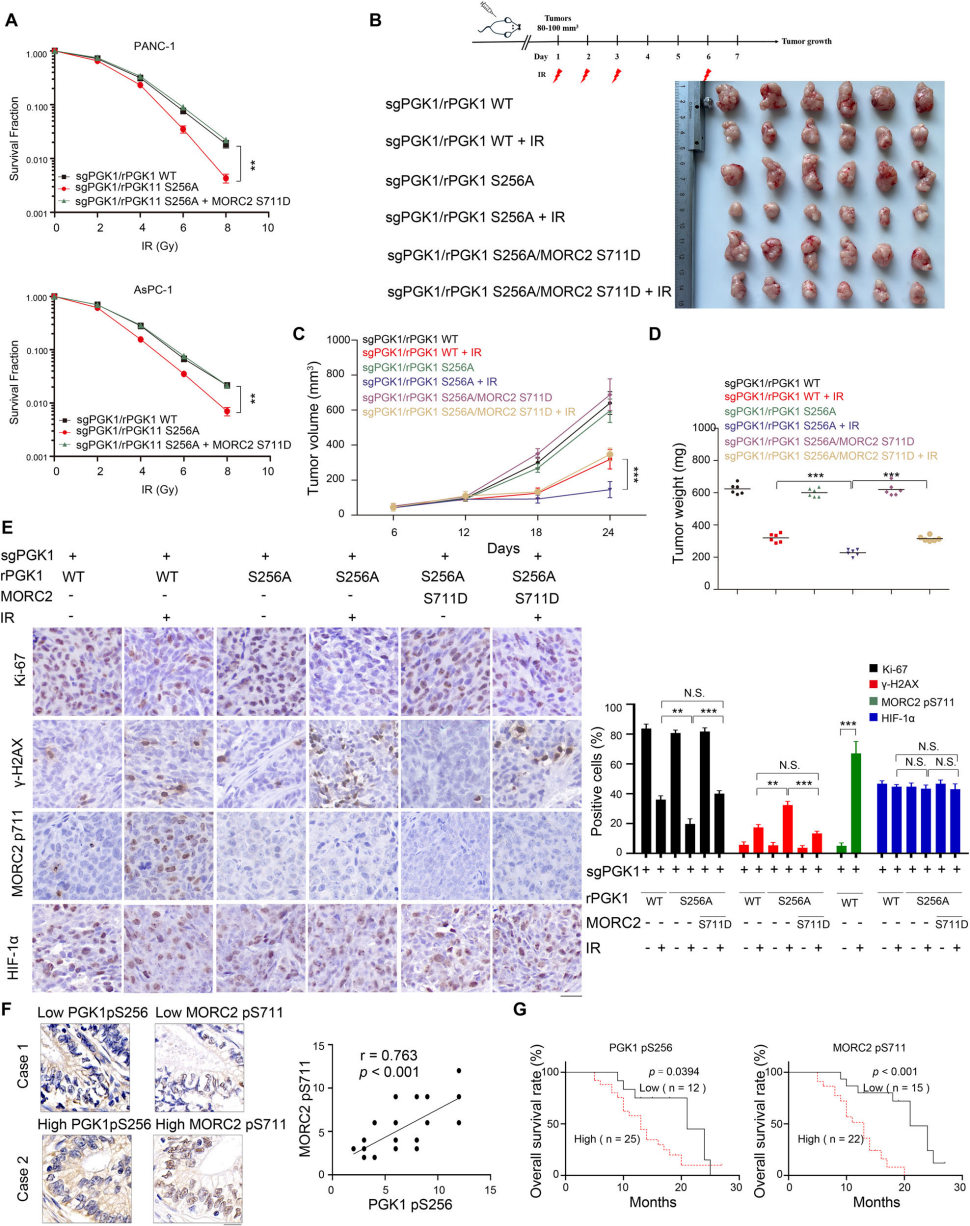
PGK1-mediated MORC2 S711 phosphorylation promotes radioresistance of PDAC. **A** PGK1 sgRNA-expressing PANC-1 and AsPC-1 cells with or without V5-MORC2 protein were stably transfected with PGK1 Flag-rPGK1 WT, S256A. Clonogenic survival assays were performed in those indicated PDAC cells with exposure to or not to IR. Data are presented as the means ± SD from three independent experiments (*n* = 3). **p < 0.01 using one-way ANOVA, followed by Bonferroni’s post hoc test. **B**–**E** PGK1 sgRNA-expressing PANC-1 cells with or without V5-MORC2 protein were stably transfected with PGK1 Flag-rPGK1 WT, S256A subcutaneously injected into nude mice (*n* = 6/group). The mice were euthanized and examined for tumor growth 24 days after injection. Images of the tumors are shown (**B**). The tumor volumes were calculated (**C**). The tumor weights were measured (**D**). Data are presented as the means ± SD for 6 mice. ***p < 0.001 using one-way ANOVA, followed by Bonferroni’s post hoc test. **E** IHC analyses of tumor samples were performed with the indicated antibodies. Ki67, γ-H2AX, MORC2 S711 phosphorylation and HIF-1α-positive cells were quantified in 10 microscopic fields. Scale bars, 20 mm. N.S., not significant for the indicated comparison, **p < 0.01 or ***p < 0.001 using one-way ANOVA, followed by Bonferroni’s post hoc test. **F** Expression of PGK1 in 37 samples of human PDAC tissues and matched adjacent normal tissues by IHC staining with the indicate antibodies. The correlation between PGK1S256 phosphorylation and MORC2 S711 phosphorylation levels was analyzed (Spearman’s correlation test; *r* = 0.763, *p* < 0.001). Representative images are shown. Scale bars, 20 μm. **G** Kaplan–Meier plots and *p*-values of the log-rank test for comparing survivals of 37 PDAC patients with high and low expression of PGK1 pS256 (*p* = 0.0394) and MORC2 pS711 (*p* < 0.001).

To examine whether PGK1-mediated radioresistance in PDAC cells depends on MORC2, we assessed the survival of PDAC cells following IR exposure. Specifically, we examined PANC-1 and AsPC-1 cells with endogenous PGK1 depletion and reconstituted expression of WT rPGK1, rPGK1 S256A, or MORC2 S711D. Compared to cells expressing WT rPGK1, those expressing rPGK1 S256A exhibited reduced colony formation ability after IR exposure (Fig. 6A). Conversely, overexpression of the MORC2 S711D phosphorylation-mimicking mutant restored colony formation in PANC-1 and AsPC-1 cells with endogenous PGK1 depletion and reconstituted expression of Flag-rPGK1 S256A (Fig. 6A).

Accordingly, we explored whether inhibiting the nonmetabolic activities of PGK1 could overcome radioresistance in PDAC using a subcutaneous tumor model in athymic nude mice. PANC-1 cells with endogenous PGK1 depletion and reconstituted expression of WT rPGK1, rPGK1 S256A, or MORC2 S711D were injected subcutaneously. Once tumors reached 80–100 mm³, mice were randomized into control and treatment groups and subjected to IR as shown in Fig. 6B. Consistent with our in vitro findings, tumors with endogenous PGK1 depletion and reconstituted expression of rPGK1 S256A exhibited significantly slower growth, reduced tumor volume (Figs. 6B, C), and tumor weight (Fig. 6D), as well as lower Ki67 expression (Fig. 6E) and higher γ-H2AX expression (Fig. 6E) compared to WT rPGK1-expressing tumors after IR. Notably, MORC2 S711D overexpression largely abolished this radiosensitivity, restoring Ki67 expression and inhibiting γ-H2AX expression (Fig. 6B–D). In addition, the orthotopic PDAC model in athymic nude mice similarly demonstrated that tumors expressing endogenous PGK1-depleted and rPGK1 S256A exhibited radiosensitivity, while MORC2 S711D overexpression largely abolished this radiosensitivity (Fig. S8A–C). Since PDAC tumors are characterized by high levels of hypoxia, and hypoxia mainly promotes radiation resistance by enhancing the expression of downstream genes through HIF-1α [38, 39]. We measured HIF-1α levels in tissues from nude mouse models to determine whether PGK1-MORC2-promoted PDAC radioresistance depends on a hypoxic mechanism. IHC results in nude mouse tissues showed that there were no significant differences in HIF-1α expression between tumors that were PGK1-depleted and reconstituted with rPGK1-S256A and tumors reconstituted with wild-type rPGK1 (Fig. 6E). Additionally, MORC2-S711D overexpression did not alter HIF-1α expression (Fig. 6E). These results suggest that PGK1-mediated MORC2-S711 phosphorylation promotes PDAC radioresistance through a hypoxia-independent mechanism. Collectively, these results indicate that PGK1-mediated phosphorylation of MORC2 at S711 is crucial for PDAC radioresistance.

We next performed IHC analyses on 37 radiotherapy-treated human PDAC specimens, demonstrating a significant positive correlation between PGK1 pS256 and MORC2 pS711 phosphorylation levels (Figs. 6F and S8D). Furthermore, elevated phosphorylation levels at both PGK1 S256 and MORC2 S711 sites showed a significant inverse correlation with overall patient survival (Fig. 6G). These results collectively underscore the critical role of PGK1-mediated MORC2 S711 phosphorylation in driving radioresistance and disease progression in PDAC.

To evaluate the pan-cancer relevance of this mechanism, we assessed whether PGK1-mediated phosphorylation of MORC2 at S711 promotes radioresistance beyond PDAC. Intriguingly, reconstituting endogenously PGK1- or MORC2-depleted HT29 colorectal cancer cells with the phosphorylation-defective mutants PGK1-S256A or MORC2-S711A, respectively, conferred heightened radiosensitivity relative to WT counterparts. Furthermore, overexpression of the MORC2-S711D restored the radiosensitive phenotype in cells subjected to endogenous PGK1 depletion and reconstituted with PGK1-S256A (Fig. S8E, F). This finding underscores the critical function of PGK1/MORC2 signaling in mediating radioresistance in colorectal cancer. Collectively, these data indicate that the PGK1/MORC2 axis not only drives radioresistance in PDAC but may exhibit broader relevance in other cancers.

## Discussion

**Fig. 7:**
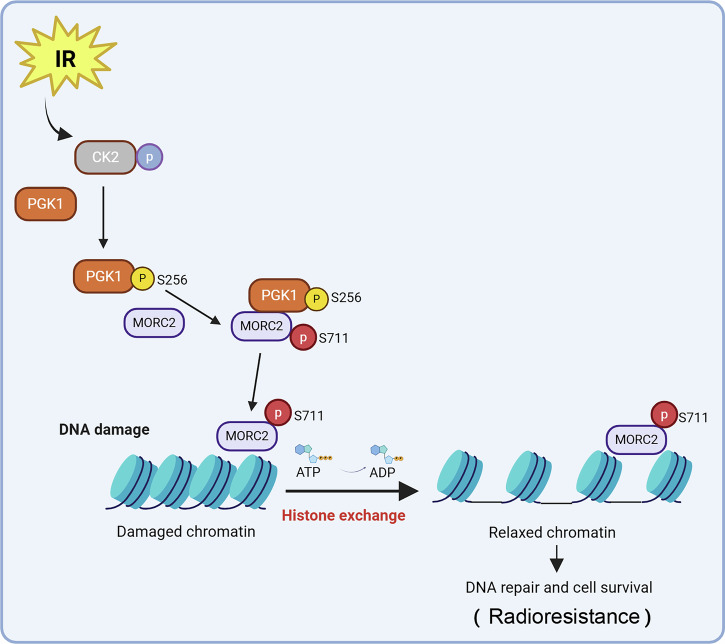
A mechanism of PGK1 mediating radiotherapy resistance. Schematic model shows that PGK1 acts as a protein kinase to phosphorylate MORC2 S711 and phosphorylated MORC2 regulates its DNA-dependent ATPase activity to facilitate chromatin remodeling, which facilitates DNA repair efficiency and were insensitive to IR.

PDAC is a highly prevalent and aggressive malignancy, with conventional chemotherapy interventions offering only marginal benefits. This situation is partly due to the aggressive nature of cancer cells, underscoring the urgent need for novel therapeutic strategies to combat this deadly disease. Recent advancements in radiotherapy have significantly improved overall survival rates for PDAC patients [40]. However, clinical research has shown variable response rates among different patient populations [41–43], highlighting the necessity for more precise treatment regimens tailored to individual patient characteristics. Our study identifies PGK1 as a potential biomarker for PDAC radioresistance and demonstrates that PGK1 overexpression enhances DNA damage repair efficiency through MORC2 phosphorylation. Inhibiting PGK1’s nonmetabolic activities sensitizes PDAC cell lines to IR by slowing DNA break repair. In summary, our findings indicate that PGK1’s nonmetabolic activities mediate DNA damage repair in PDAC and contribute to radioresistance (Fig. 7).

Metabolism, a fundamental biological process, comprises a series of chemical reactions catalyzed by metabolic enzymes. These enzymes are responsible for specific chemical reactions on metabolites under strict regulation at specific subcellular locations in metabolic cascades. Our research group and others have demonstrated that several metabolic enzymes possess nonmetabolic activities, which play critical roles in various cellular activities, including gene expression, redox homeostasis, anabolism, catabolism, and DNA repair. These nonmetabolic activities can be divided into two categories. First, metabolic enzymes that translocate from their original subcellular compartments to different organelles, where their metabolite production directly modifies or regulates other proteins. For instance, mitochondrial α-ketoglutarate dehydrogenase (α-KGDH) translocates to the nucleus and produces succinyl-coenzyme A (CoA), which is used by histone acetyltransferase 2 A (KAT2A) to succinylate histone H3 on lysine 79, around the transcription-starting sites of genes [3, 44]. Second, metabolic enzymes that use non-metabolites as substrates to catalyze reactions distinct from their originally characterized metabolic functions. For example, fructose-1,6-bisphosphatase 1 (FBP1) acts as a protein phosphatase to dephosphorylate TERT S227, inducing cell senescence, and its deficiency promotes tumor growth in hepatocellular carcinoma [45]. The non-canonical functions of metabolic enzymes make them promising therapeutic targets for cancer treatment.

PGK1, a key enzyme in the glycolytic pathway, generates ATP and maintains energy homeostasis in tumor cells. Several groundbreaking studies have confirmed PGK1’s roles in tumor initiation and progression through various non-canonical pathways. It has been shown that CK2 phosphorylates nuclear PGK1 at S256, enabling PGK1 to interact with CDC7 under EGFR activation conditions. CDC7-bound PGK1 converts ADP to ATP, thereby relieving ADP’s inhibition of CDC7-ASK activity and promoting DNA replication and tumorigenesis [16]. In this study, we demonstrate that IR-activated CK2 phosphorylates PGK1 at S256. This phosphorylation promotes PGK1-MORC2 interaction. PGK1 then acts as a protein kinase to phosphorylate MORC2 at S711, enhancing MORC2’s DNA-dependent ATPase activity. This facilitates chromatin remodeling, improves DNA repair efficiency, and confers IR insensitivity (Fig. 7). In summary, we report a novel function of the PGK1-MORC2 axis in the DNA damage response, mediated through phosphorylation-dependent, ATPase-coupled chromatin remodeling (Fig. 5). PGK1’s nonmetabolic activity-driven activation of MORC2 stimulates PDAC radioresistance (Fig. 6). Thus, inhibiting PGK1 S256 phosphorylation may enhance pancreatic cancer’s radiotherapy sensitivity. Importantly, PGK1’s metabolic and nonmetabolic activities depend on different sites. Further investigation into the mechanisms regulating PGK1 function is required to better understand how PGK1 signaling is maintained in tumor cells. Furthermore, we observed that the PGK1-mediated MORC2-S711 phosphorylation also promotes radioresistance in colorectal cancer. These pan-cancer observations suggest that the PGK1/MORC2 pathway represents a shared mechanism of radioresistance across multiple solid tumors. This warrants further investigation into its therapeutic potential as a pan-cancer target for radiosensitization. Future studies should comprehensively assess PGK1/MORC2 expression patterns, functional roles, and therapeutic efficacy across diverse IR-treated malignancies. Finally, while our integrated in vitro and in vivo approaches (employing PDAC cell lines and subcutaneous and orthotopic models in nude mice) provide mechanistic insights, these systems can only partially recapitulate the complexity of the human tumor microenvironment. Future investigations utilizing patient-derived specimens and immunocompetent murine PDAC models are warranted to delineate microenvironmental influences on radioresistance.

Neoadjuvant therapy has garnered attention for treating resectable and borderline resectable PDAC, aiming to increase resection rates and improve survival. However, multicenter phase III randomized controlled trials in adjuvant and neoadjuvant settings have yielded inconsistent results regarding radiation therapy’s benefits [46, 47]. To address this challenge, studies have emphasized escalating radiation dosages to enhance local control and 5-year overall survival rates [48, 49]. Unfortunately, PDAC’s relative insensitivity to radiotherapy and the poor tolerance of surrounding pancreatic tissues limit the radiation dose. Improving PDAC’s radiotherapy sensitivity holds great clinical significance. MORC2 plays a crucial role in radiation resistance by regulating the DNA damage response pathway [22]. Unperturbed heterochromatin acts as a barrier to DNA damage response and repair processes, necessitating its alleviation to enable DSB repair [50]. Recent studies have shown that IR activates PAK1 kinase, which interacts with and phosphorylates MORC2 on serine 739. Phosphorylated MORC2 associates with chromatin and facilitates ATPase-dependent chromatin relaxation in response to DNA damage, reorganizing higher-order chromatin structure through induced chromatin relaxation [22]. However, the mechanisms regulating MORC2 activity remain unclear. We demonstrate that IR activates CK2, which phosphorylates PGK1 at S256 (Fig. 3). PGK1, acting as a protein kinase, phosphorylates MORC2 at S711, enhancing MORC2 activity (Figs. 4 and 5). A better understanding of the mechanisms underlying MORC2-induced radioresistance is crucial for developing more effective combination therapy strategies incorporating radiation.

Notably, PDAC exhibits significant molecular heterogeneity, with 4–7% of patients harboring pathogenic BRCA1 or BRCA2 (BRCA1/2) mutations. Deficiency in BRCA1/2 function impairs the homologous recombination repair (HRR) pathway responsible for repairing DSBs. This defect leads to genomic instability and ultimately cell death [51]. Given that DSBs represent the most biologically significant lesions induced by IR, and defective DSB repair confers cellular radiosensitivity, BRCA1/2-mutant cancer patients were hypothesized to derive substantial benefit from radiotherapy. However, clinical evidence supporting this hypothesis remains inconsistent and inconclusive [52]. Our study focuses on PGK1-mediated radioresistance in BRCA1/2 wild-type PDAC (PANC-1, AsPC-1), leaving PGK1 inhibition’s impact on BRCA1/2-mutant PDAC radiosensitivity unexplored—a critical future direction for advancing PDAC precision therapy.

The optimal treatment approach in the neoadjuvant setting for borderline resectable and resectable PDAC remains a topic of ongoing debate. Here, we reveal a novel mechanism underlying PDAC radioresistance, where PGK1-dependent MORC2 S711 phosphorylation regulates the DNA damage response pathway. Our study provides a molecular basis for developing therapeutic strategies to overcome radioresistance in PDAC patients with aberrant PGK1 nonmetabolic activity.

## Supplementary information

Supplementary figure legend

Supplementary materials

Supplementary Figure 1

Supplementary Figure 2

Supplementary Figure 3

Supplementary Figure 4

Supplementary Figure 5

Supplementary Figure 6

Supplementary Figure 7

Supplementary Figure 8

Supplementary original western blots

## Data Availability

Further information can be obtained through the lead contact, Dong Yan (yd15yt88@163.com**)**.
