# Supplementary material for: The glycolytic enzyme PGK1 phosphorylates MORC2 to Confer radioresistance in pancreatic ductal adenocarcinoma
Source: Cell Death Dis. 2025 Nov 10;16(1):824. doi: 10.1038/s41419-025-08177-9 (PMC12603276; doi:10.1038/s41419-025-08177-9)
Supplement: Supplementary file 1 — Supplementary figure legend [file 41419_2025_8177_MOESM1_ESM.docx]

**Supplementary Figure S1. The schematic diagram of PGK1 activity**

(A) The schematic diagram of PGK1 metabolic enzyme activity and protein kinase activity.

**Supplementary Figure S2. PGK1 is highly expressed in PDAC specimens and indicates poorer prognosis, related to Figure 1**

(A) The expression of PGK1 analysed by GEPIA. Red colour means PDAC tissues and grey colour means normal tissues. **p* < 0.05.

(B-C) DFS (HR = 2.7, *p* = 0.0057) and Survival curves of OS (HR = 2, *p* = 0.023) in PDAC in GEPIA database.

**Supplementary Figure S3.** **PGK1 facilitates resistance to radiotherapy in PDAC by inhibiting the DNA damage response, related to Figure 2**

(A) PGK1 was overexpressed in the indicated cells. Immunoblot analyses were performed using the indicated antibodies.

(B) PANC-1 and AsPC-1 cells with or without PGK1 overexpression were exposed to IR or left untreated. Clonogenic survival assays were performed on the indicated cells. Data represent means ± SD from three independent experiments (n = 3). **p* < 0.05 or ****p* < 0.001 using unpaired Student’s t test.

(C-D) The PANC-1 and AsPC-1 cells were exposure to or not to IR. RT-PCR (C) and immunoblot analyses (D) were performed. N.S., not significant for the indicated comparison. (E) PANC-1 and AsPC-1 cells with or without PGK1 overexpression were exposed to 4 Gy IR for designated time points. Immunoblot analyses were performed using the indicated antibodies.

(F-G) Indicated cells were cultured in the presence or absence of DOX (0.4 μM) for the indicated time. Cell proliferation was examined using a CCK- 8 assay (F). Data are presented as the means ± SD from three independent experiments (n = 3). ***p* < 0.01 using one-way ANOVA, followed by Bonferroni’s post hoc test (E). Immunoblot analyses were performed using the indicated antibodies (G).

**Supplementary Figure S4. PANC-1 cells were pretreated with or without inhibitors targeting IR stress-responsive kinases, related to Figure 3**

(A) PANC-1 cells were pretreated with or without LY294002(30 μM), U0126 (20 µM), SP600125 (25 μM), TBB(10 μM) for 1 h before exposure to IR (4 Gy) for 6 h. Immunoblot analyses were performed with the indicated antibodies.

**Supplementary Figure S5. PGK1 phosphorylates MORC2 at S711, related to Figure 4**

(A) *In vitro* phosphorylation analyses were performed by mixing purified His-CK2 with the indicated purified His-PGK1 and GST or GST-MORC2 protein in the presence of ATP-γ-S. Immunoblot analyses were performed with the indicated antibodies.

(B) *In vitro* phosphorylation analyses were performed by mixing purified His-CK2 with the indicated purified His-MORC2 and GST-PGK1 protein. Immunoblot analyses were performed with an anti-MORC2 pS711 antibody in the presence or absence of a phospho-S711 blocking peptide.

(C) *In vitro* phosphorylation analyses were performed by mixing purified His-CK2 with the indicated purified His-MORC2 and GST-WT PGK1 or GST-PGK1 T378P protein in the presence of ATP-γ-S. Immunoblot analyses were performed with the indicated antibodies.

(D) *In vitro* phosphorylation analyses were performed by mixing purified His-CK2 with the indicated purified His-MORC2 and GST-PGK1 protein (0 - 500 ng) in the presence of ATP-γ-S. Immunoblot analyses were performed with the indicated antibodies.

(E) Expression of WT Flag-rPGK1 and Flag-rPGK1 S256A was reconstituted in endogenous PGK1-depleted PANC-1 and AsPC-1 cells. Immunoblot analyses were performed with the indicated antibodies.

**Supplementary Figure S6. PGK1-phosphorylated MORC2 S711 promotes its DNA-Dependent ATPase activity, related to Figure 5**

(A) PGK1 sgRNA-expressing PANC-1 cells with or without Flag-MORC2 were stably transfected with V5-rPGK1 WT, S256A, and were exposure to IR (4 Gy). After 1 hr of IR treatment, nuclear extracts were immunoprecipitated with Flag-tagged agarose beads and subjected to ATPase assays using 100 ng of double-stranded plasmid DNA.

(B) Expression of WT Flag-rMORC2, Flag-rMORC2 S711A or Flag-rMORC2 D68A was reconstituted in endogenous MORC2-depleted PANC-1 and AsPC-1 cells. Immunoblot analyses were performed with the indicated antibodies.

(C) The indicated cells with endogenous MORC2 depletion and reconstituted expression of Flag-rMORC2 or Flag-rMORC2 S711A were exposure to or not to IR (4 Gy). The indicated cells were harvested at different time points for IF analyses. Data are presented as the means ± SD from three independent experiments (n = 3). **p* < 0.05 using unpaired Student’s t test. Scale bars, 10 μm.

(D) PANC-1 and AsPC-1 cells with endogenous MORC2 depletion and reconstituted expression of Flag-rMORC2 or Flag-rMORC2 S711A were exposure to IR (4 Gy). Salt solubilization assays were performed and the core histones in salt soluble fractions were detected by immunoblotting using the indicated antibodies.

**Supplementary Figure S7. PGK1 S256 phosphorylation does not affect PGK1's metabolic enzyme activity, but can modulate the DNA-dependent ATPase activity of MORC2, related to Figure 5**

(A-E) PANC-1 and AsPC-1 cells transduced with control sgRNA or PGK1 sgRNA-3 vectors, followed by reconstitution with WT rPGK1 or rPGK1 S256A, were cultured.

ECAR and OCR (A),  cell cycle analysis (B) and  cell viability (C) were measured in these cells.

(D) The indicated cell groups were exposed or not exposed to IR, followed by clonogenic survival assays.

(E) The indicated cell groups were exposed to IR, followed by MNase assays.

(A-D) Data are presented as the means ± SD from three independent experiments (n = 3). N.S., no significant difference ,***p* < 0.01 or ****p* < 0.001 using one-way ANOVA, followed by Bonferroni’s post hoc test.

**Supplementary Figure S8. PGK1-mediated MORC2 S711 phosphorylation promotes radioresistance,** **related to Figure 6**

(A-C) PGK1 sgRNA-expressing PANC-1 cells with or without V5-MORC2 S711D were stably transfected with PGK1 Flag-rPGK1 WT, S256A orthotopically injected into nude mice (n = 6/group). The mice were euthanized and examined for tumor growth 24 days after injection. Images of the tumors are shown (A). The tumor volumes were calculated (B). The tumor weights were measured (C). Data are presented as the means ± SD for 6 mice. ***p < 0.001 using one-way ANOVA, followed by Bonferroni’s post hoc test.

(D) The specificities of the PGK1 S256 and MORC2 S711 phosphorylation antibodies were validated using IHC analyses with corresponding specific blocking phosphorylation peptides. Scale bars: 20 μm.

(E) PGK1 was depleted in the indicated cells by expressing PGK1 sgRNA (left). Expression of WT Flag-rPGK1 and Flag-rPGK1 S256A was reconstituted in endogenous PGK1-depleted HT29 cells (middle). Expression of WT Flag-rMORC2 or Flag-rMORC2 S711A was reconstituted in endogenous MORC2-depleted HT29 cells (right). Immunoblot analyses were performed with the indicated antibodies.

(F) The indicated HT29 cells were exposed to or not to IR. Clonogenic survival assays were performed in indicated cells. Data are presented as the means ± SD from three independent experiments (n = 3). ***p* < 0.01 or ****p* < 0.001 using one-way ANOVA, followed by Bonferroni’s post hoc test.
