# Supplementary material for: The glycolytic enzyme PGK1 phosphorylates MORC2 to Confer radioresistance in pancreatic ductal adenocarcinoma
Source: Cell Death Dis. 2025 Nov 10;16(1):824. doi: 10.1038/s41419-025-08177-9 (PMC12603276; doi:10.1038/s41419-025-08177-9)
Supplement: Supplementary file 2 — Supplementary materials [file 41419_2025_8177_MOESM2_ESM.docx]

**The Glycolytic Enzyme PGK1 Phosphorylates MORC2 to Confer Radioresistance in**

**Pancreatic Ductal Adenocarcinoma**

Yingying Tong, Xin Liu, Qian Liu, Jing Wang, Yaoxian Xiang, Kangjie Wang, Zeyu Zhao, Ke Zhu, Lijun Yang, Li Wang, Dong Guo, Zhimin Lu, Dong Yan

**Supplemental Tables**

**Table S1.** **Primers for DNA constructs and mutagenesis**

| **Primer** | **Sequence** | **Base number** |
| --- | --- | --- |
| PGK1 S256A-F | CCTCTTCATCAAACAGAGCAGTGCCAATC  TCCATGTT | 37 |
| PGK1 S256A-R | AACATGGAGATTGGCACTGCTCTGTTTGA  TGAAGAGG | 37 |
| PGK1 T378P -F | GGTGGAGACACTGCCCCTTGCTGTGCCA  AAT | 31 |
| PGK1 T378P -R | ATTTGGCACAGCAAGGGGCAGTGTCTC  CACC | 31 |
| MORC2 S711A-F | TGATGACTTTGGGAGCAGGAACCTCCCG  AGG | 31 |
| MORC2 S711A-R | CCTCGGGAGGTTCCTGCTCCCAAAGTCA  TCA | 31 |
| MORC2 S711D-F | GATGACTTTGGGATCAGGAACCTCCCGAG  GGCTC | 34 |
| MORC2 S711D-R | GAGCCCTCGGGAGGTTCCTGATCCCAAAG  TCATC | 34 |
| MORC2 D68A-F | ATTTATGCTTTGCTTTTTGGCTGATGGAGC  AGGAATGGATC | 41 |
| MORC2 D68A-R | GATCCATTCCTGCTCCATCAGCCAAAAAGC  AAAGCATAAAT | 41 |
| PGK1-sg#1 | CAGGGATGTTCTGTTCTTGA | 20 |
| PGK1-sg#2 | ACCTCTGGTTGTTTGTTATC | 20 |
| PGK1-sg#3 | CAGGGATGTTCTGTTCTTGA | 20 |
| MORC2-sgRNA | TCAGGGGCTCAATGCGCATT | 20 |

**Table S2. Primers for qPCR analysis**

| **Primer** | **Sequence** | **Base number** |
| --- | --- | --- |
| *MORC2*-F | TATGCCGCTGTGCTCTAT | 18 |
| *MORC2*-R | TTCTTCACCTCCTGCTCC | 18 |
| *GAPDH*-F | GGAGCGAGATCCCTCCAAAAT | 21 |
| *GAPDH*-R | GGCTGTTGTCATACTTCTCATGG | 23 |

**Table S3.**

**Clinicopathological factors in 60 PDAC patients**

| **Characteristics** | | **n = 60** |
| --- | --- | --- |
| **Age** |  |  |
| ≤ 60 |  | 31 |
| > 60 |  | 29 |
| **Sex** |  |  |
| Male |  | 40 |
| Female |  | 20 |
| **Greatest tumor diameter (cm)** | | |
| ≤ 4 |  | 36 |
| > 4 |  | 24 |
| **Lympy node involvement** | | |
| Negative |  | 33 |
| Positive |  | 27 |
| **Histologic grade** | | |
| ≤ II |  | 38 |
| > II |  | 22 |
| **Stage** |  |  |
| ≤ II |  | 51 |
| > II |  | 9 |

**Clinicopathological factors in 37 PDAC patients**

| **Characteristics** | | **n = 37** |
| --- | --- | --- |
| **Age** |  |  |
| ≤ 60 |  | 17 |
| >60 |  | 20 |
| **Sex** |  |  |
| Male |  | 19 |
| Female |  | 18 |
| **Greatest tumor diameter (cm)** | | |
| ≤ 4 |  | 20 |
| > 4 |  | 17 |
| **Lympy node involvement** | | |
| Negative |  | 6 |
| Positive |  | 31 |
| **Histologic grade** | | |
| ≤ II |  | 20 |
| > II |  | 17 |

**Table S4. The antibodies and reagents used in this study**

| REAGENT or RESOURCE | SOURCE | IDENTIFIER |
| --- | --- | --- |
| **Antibodies** | | |
| Rabbit monoclonal anti-V5 | abcam | Cat#4ab206566 |
| Mouse monoclonal anti-β-actin | abcam | Cat#4ab8224 |
| Rabbit monoclonal anti-GST | abcam | Cat# ab111947 |
| Mouse monoclonal anti-Flag | Sigma-Aldrich | Cat#F1804 |
| Rabbit monoclonal anti-p-ERK1/2 | Cell Signaling Technology | Cat# 4370 |
| Rabbit monoclonal anti-ERK1/2 | Cell Signaling Technology | Cat#4695 |
| Rabbit monoclonal anti-c-Jun pS73 | Cell Signaling Technology | Cat# 3270 |
| Rabbit monoclonal anti-c-Jun | Cell Signaling Technology | Cat#9165; |
| Rabbit monoclonal anti-p-AKT | Cell Signaling Technology | Cat#4060 |
| Rabbit monoclonal anti-AKT | Cell Signaling Technology | Cat#4685 |
| Rabbit monoclonal  anti-PGK1 | abcam | Cat#ab199438 |
| Mouse Monoclonal anti-PGK1 | absin | Cat#abs113676 |
| Rabbit monoclonal anti-H2AX | Abcam | Cat#ab229914 |
| Rabbit monoclonal anti-γ H2AX | Abcam | Cat#ab243906 |
| Rabbit monoclonal anti-Ki67 | Abcam | Cat#ab92742 |
| Rabbit monoclonal anti-MORC2 | abcam | Cat#ab315903 |
| Rabbit polyclonal to CK2 | Abcam | Cat#ab10466 |
| Rabbit monoclonal anti-Vinculin | Abcam | Cat#ab207440 |
| Rabbit monoclonal anti-H2A | Abcam | Cat#ab177308 |
| Mouse monoclonal anti-H2B | Abcam | Cat#ab52484 |
| Rabbit polyclonal to anti-H3 | Abcam | Cat#ab1791 |
| Rabbit monoclonal to H4 | Abcam | Cat#ab177840 |
| Rabbit polyclonal to anti-HNRNPM | Absin | Cat#abs102112 |
| Rabbit monoclonal anti-Thiophosphate ester | Abcam | Cat#ab92570 |
| Rabbit monoclonal to Phospho -(Ser/Thr) Phe | Abcam | Cat#ab300625 |
| Rabbit polyclonal anti-Phospho-α-Catenin(Ser641) | Abxin | Cat#abs172377 |
| Rabbit polyclonal anti-PGK1 pS256 | Signalway Biotechnology | N/A |
| Rabbit polyclonal anti-MORC2 pS711 | Signalway Biotechnology | N/A |
| Biological samples | | |
| Human PDAC tissues | Beijing Luhe Hospital,  Capital Medical University | N/A |
| **Reagents** | | |
| Anti-FLAG M2 Beads | Sigma-Aldrich | Cat#M8823 |
| Flag Peptide | Sigma-Aldrich | Cat#F4799 |
| Protease Inhibitor Cocktail | Sigma-Aldrich | Cat#5892970001 |
| Normal rabbit IgG | Santa Cruz Biotechnology | Cat#sc-2027 |
| Normal mouse IgG | Santa Cruz Biotechnology | Cat#sc-2025 |
| ATP-γ-S | Abcam | Cat#ab138911 |
| MG-132 | Abcam | Cat#ab141003 |
| DAPI | Thermo Fisher | R37606 |
| SP600125 | Selleck | Cat#S1460 |
| TBB | Selleck | Cat#S5265 |
| U0126 | Selleck | Cat#S1102 |
| LY294002 | Selleck | Cat#S1105 |
| P^32^-ATP | Perkin Elmer | Cat#NEG002A500UC |
| DMEM medium | Corning | Cat#10013CV |
| RPMI medium | Corning | Cat#11875093 |
| FBS | Gibco | Cat#26140079 |
| Puromycin | Gibco | Cat#A11138-03 |
| Kinase Buffer | Cell Signaling Technology | Cat**#**9802 |
| Cell Counting Kit-8 | MCE | Cat#HY-K0301 |
| PI/RNase Staining Buffer | BD Biosciences | Cat#550825 |
| Micrococcal Nuclease | Sigma-Aldrich | Cat#N3755 |
| Doxorubicin | Selleck | Cat#E2516 |
| Polybrene | Sigma | Cat#TR-1003-G |
| Critical commercial assays | | |
| Comet Assay Kit | AmyJet Scientific Inc | Cat#STA-350 |
| Phosphatase assay kit | Santa Cruz Biotechnology | Cat#MAK307 |
| Mut Express II Fast Mutagenesis Kit V2 | Vazyme | Cat#C214-01 |
| XF Cell Mito Stress Test Kit | Agilent | Cat#103015-100 |
| XF Glycolysis Stress Test Kit | Agilent | Cat#103020-100 |
| VECTASTAIN ABC kit | whatman | Cat#PK-6100 |
