# Supplementary figures and images for: The glycolytic enzyme PGK1 phosphorylates MORC2 to Confer radioresistance in pancreatic ductal adenocarcinoma
Source: Cell Death Dis. 2025 Nov 10;16(1):824. doi: 10.1038/s41419-025-08177-9 (PMC12603276; doi:10.1038/s41419-025-08177-9)

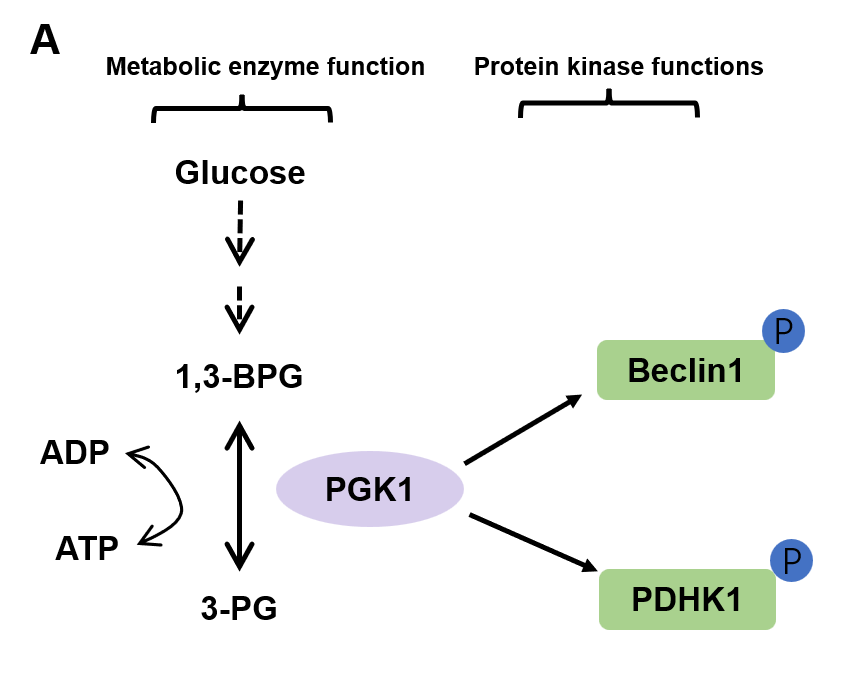

Supplement: Supplementary file 3 — Supplementary Figure 1 [file 41419_2025_8177_MOESM3_ESM.tif]

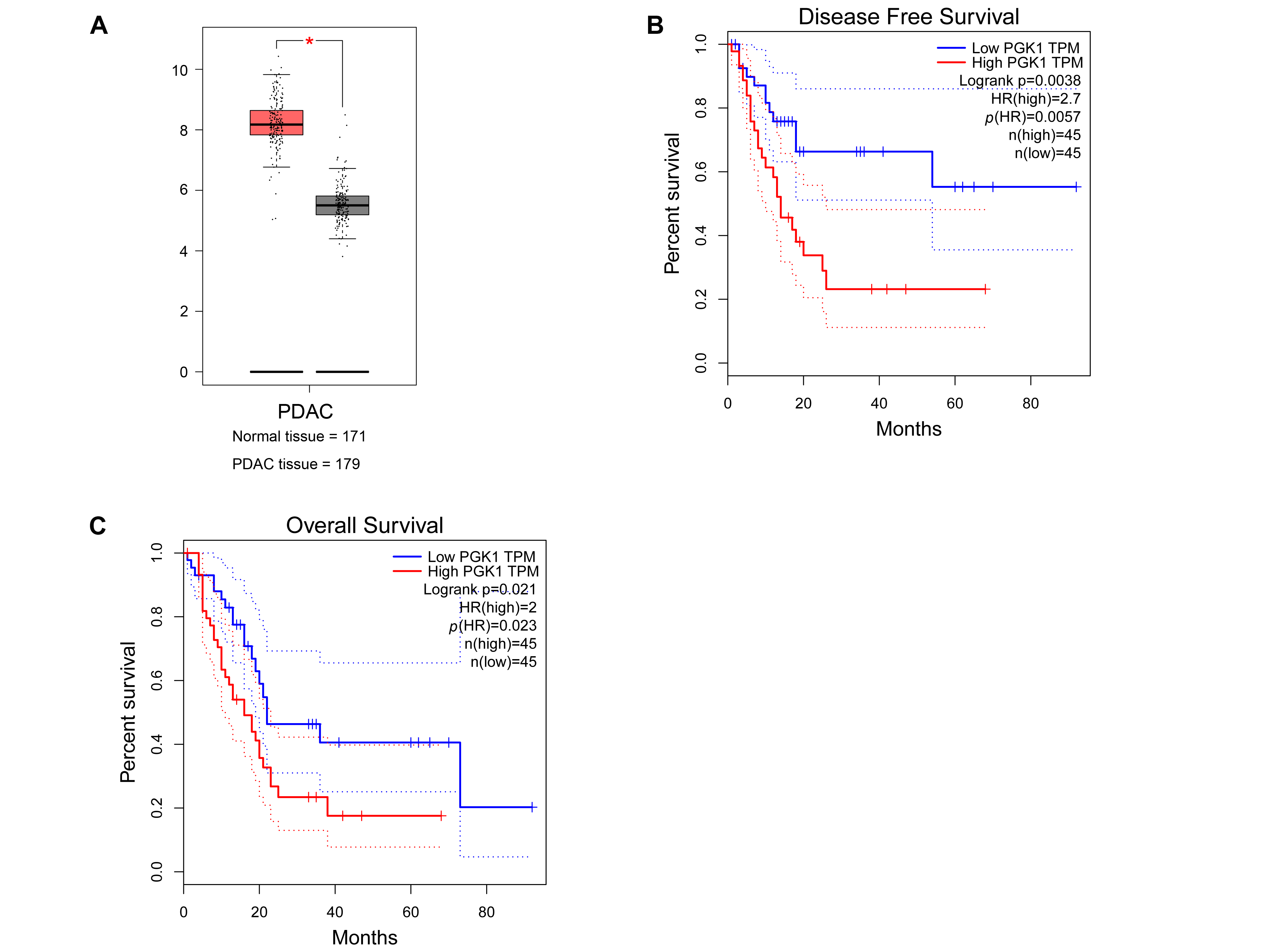

Supplement: Supplementary file 4 — Supplementary Figure 2 [file 41419_2025_8177_MOESM4_ESM.tif]

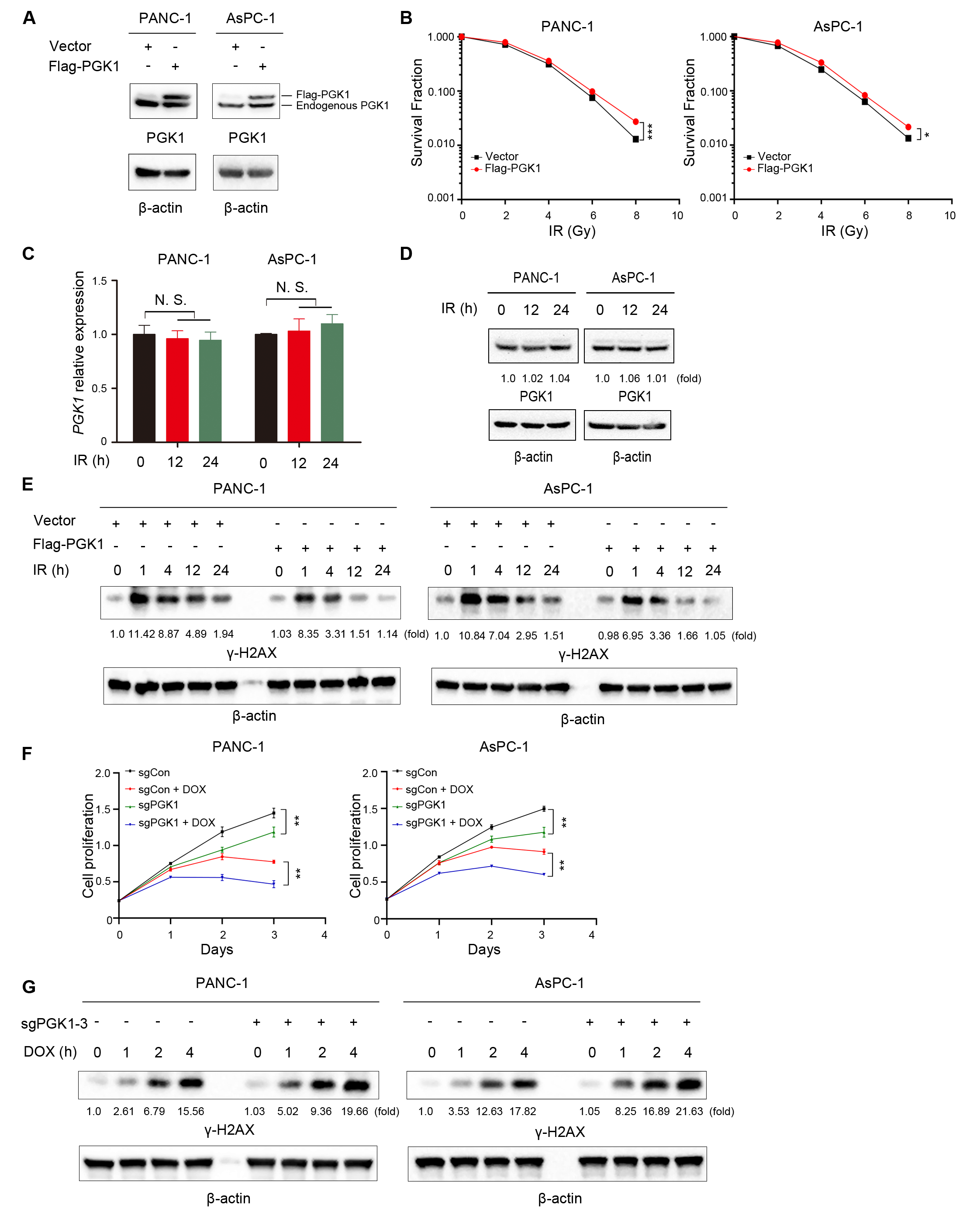

Supplement: Supplementary file 5 — Supplementary Figure 3 [file 41419_2025_8177_MOESM5_ESM.tif]

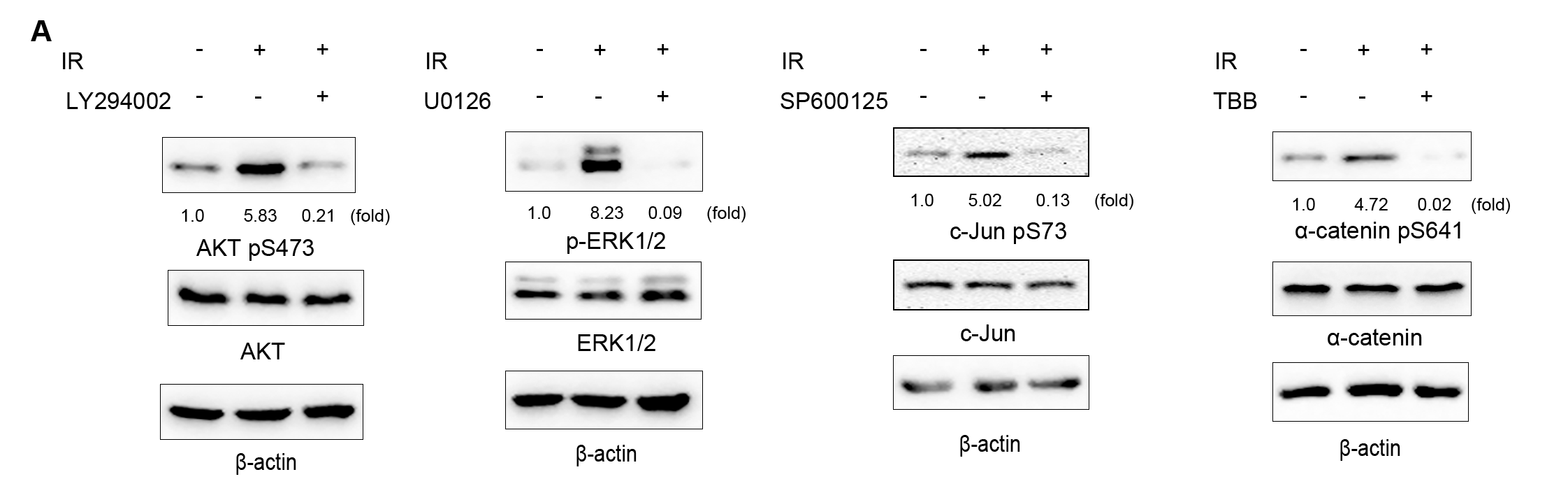

Supplement: Supplementary file 6 — Supplementary Figure 4 [file 41419_2025_8177_MOESM6_ESM.tif]

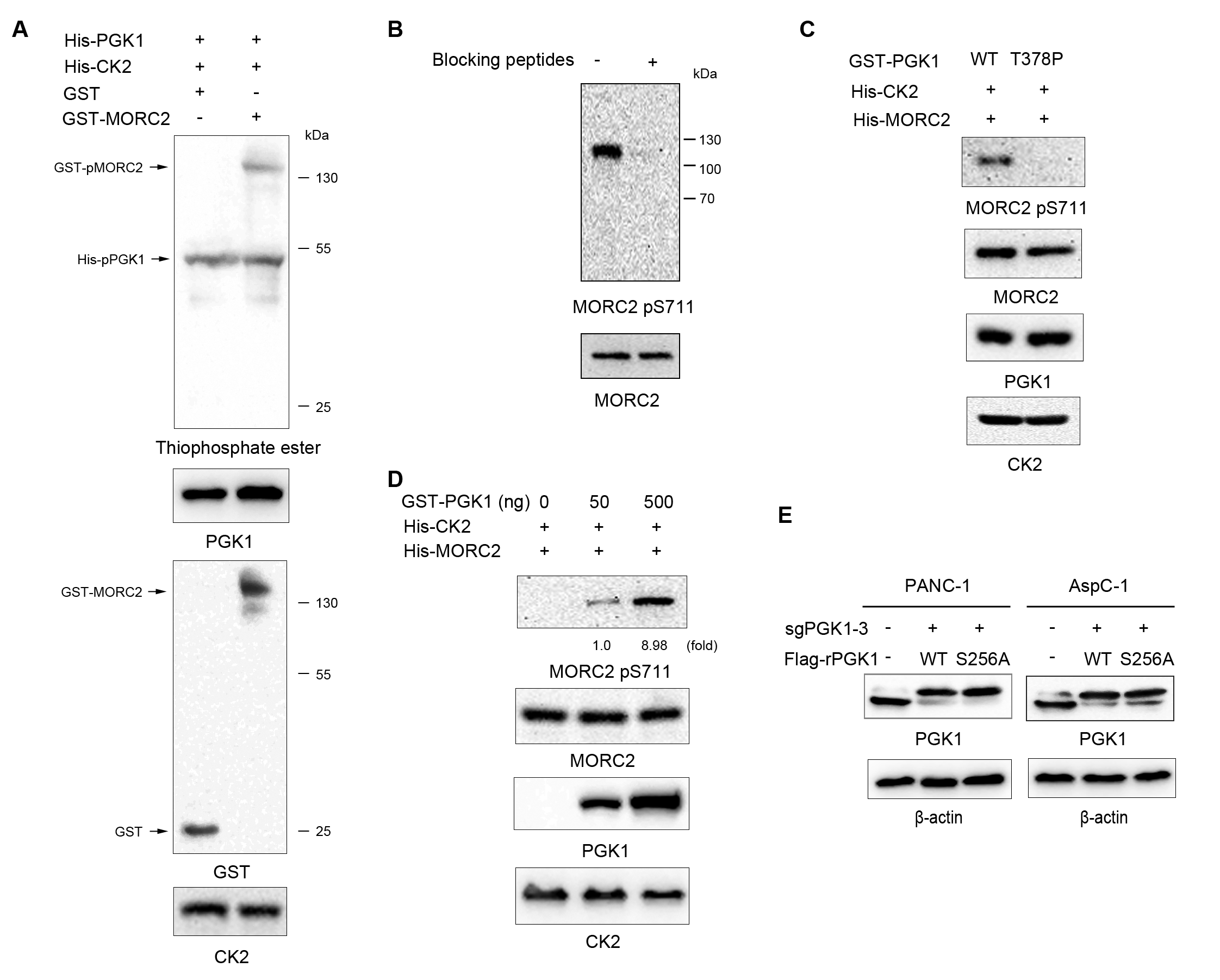

Supplement: Supplementary file 7 — Supplementary Figure 5 [file 41419_2025_8177_MOESM7_ESM.tif]

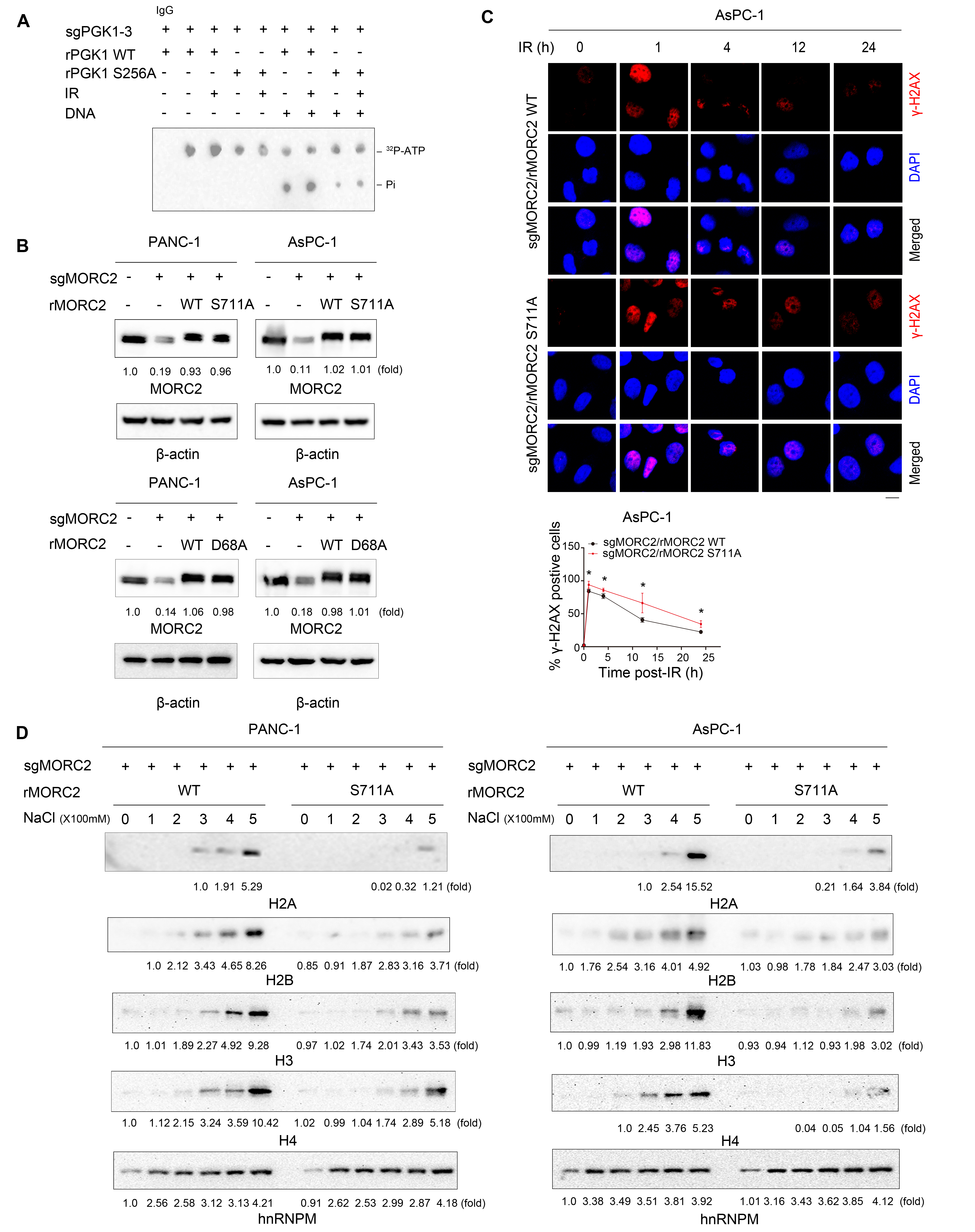

Supplement: Supplementary file 8 — Supplementary Figure 6 [file 41419_2025_8177_MOESM8_ESM.tif]

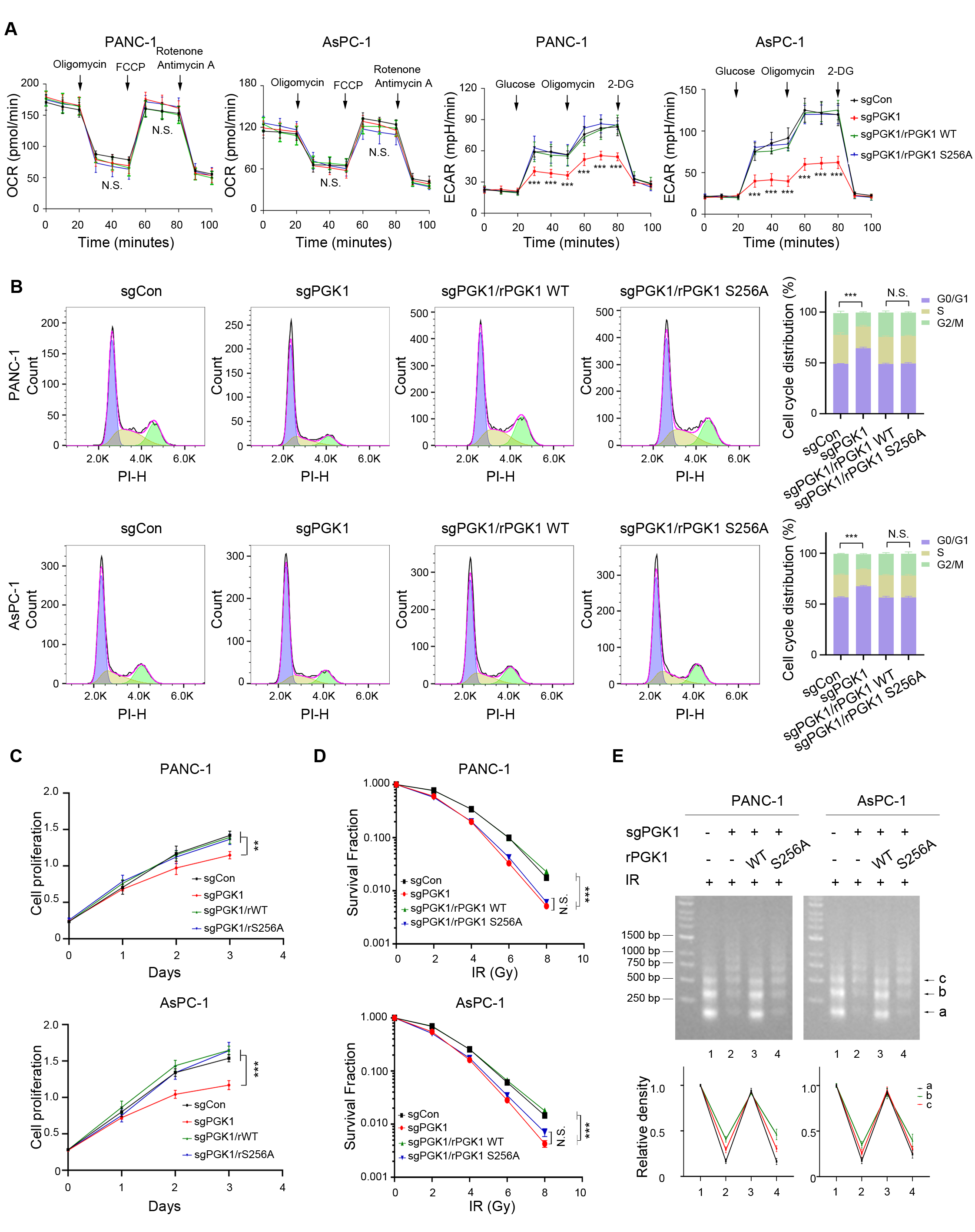

Supplement: Supplementary file 9 — Supplementary Figure 7 [file 41419_2025_8177_MOESM9_ESM.tif]

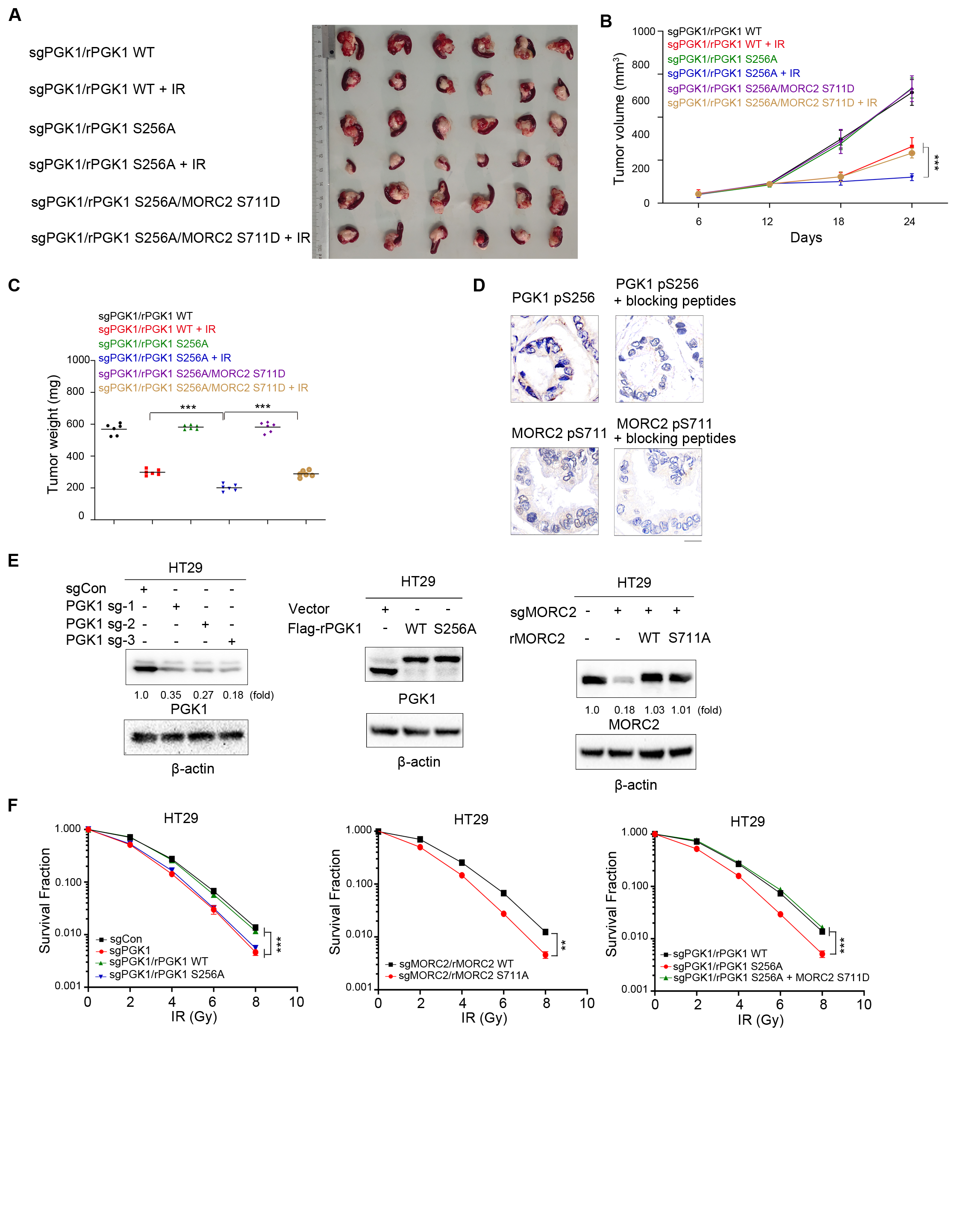

Supplement: Supplementary file 10 — Supplementary Figure 8 [file 41419_2025_8177_MOESM10_ESM.tif]
