# Supplementary material for: The glycolytic enzyme PGK1 phosphorylates MORC2 to Confer radioresistance in pancreatic ductal adenocarcinoma
Source: Cell Death Dis. 2025 Nov 10;16(1):824. doi: 10.1038/s41419-025-08177-9 (PMC12603276; doi:10.1038/s41419-025-08177-9)
Supplement: Supplementary file 11 — Supplementary original western blots [file 41419_2025_8177_MOESM11_ESM.pdf]

Supplemental Material: Original Western Blots

Relevant areas for cropped blots in the main and Supplementary figures are shown.

Fig. 1

E

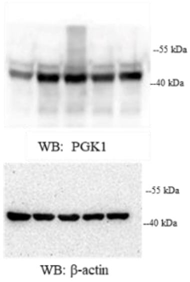

Fig. 2

A

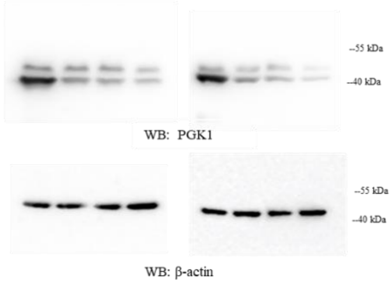

Fig. 3

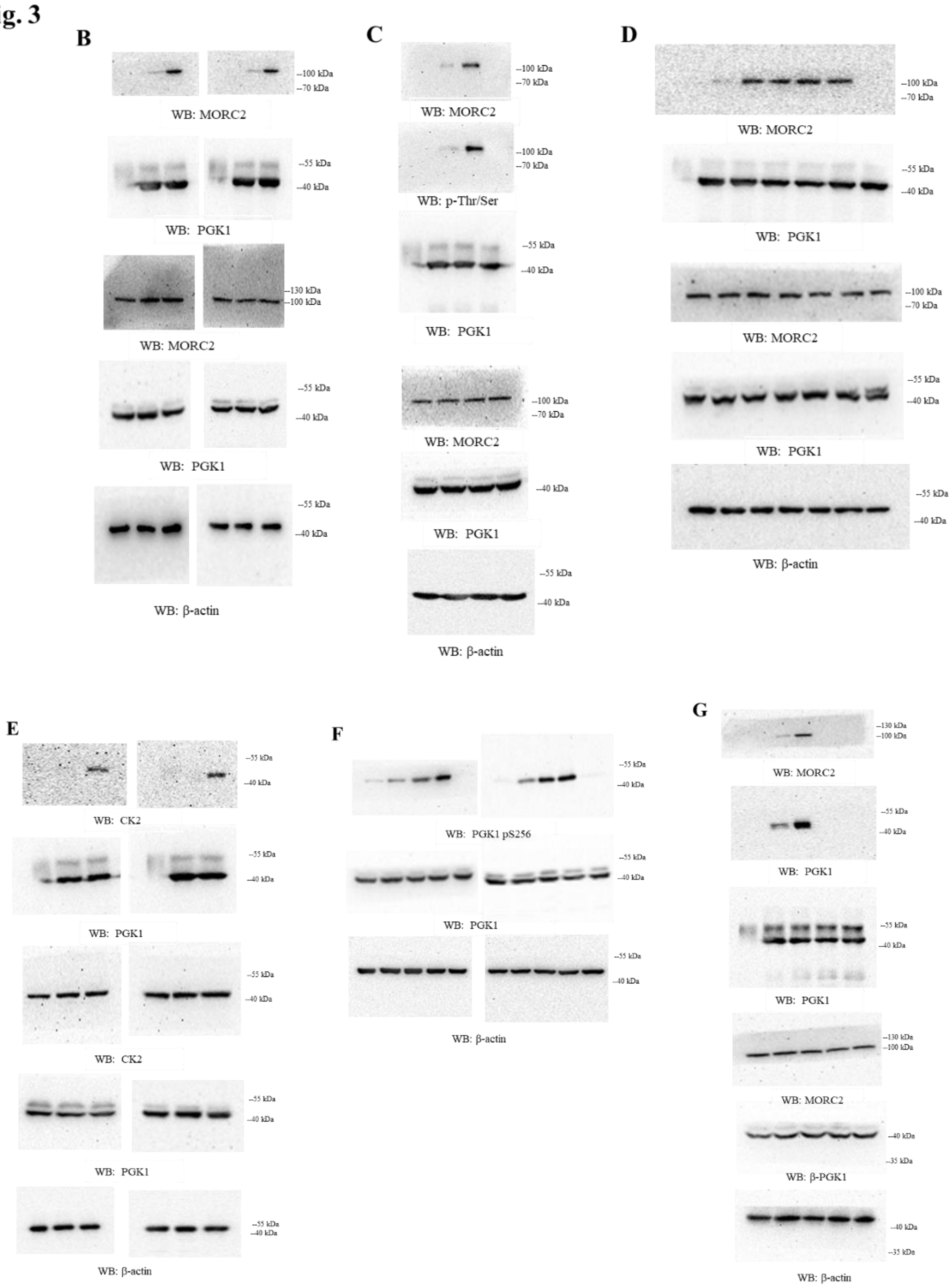

B
100 kDa
70 kDa
WB: MORC2
55 kDa
40 kDa
WB: PGK1
130 kDa
100 kDa
WB: MORC2
55 kDa
40 kDa
WB: PGK1
55 kDa
40 kDa
WB: β-actin
C
100 kDa
70 kDa
WB: MORC2
100 kDa
70 kDa
WB: p-Thr/Ser
55 kDa
40 kDa
WB: PGK1
100 kDa
70 kDa
WB: MORC2
40 kDa
WB: PGK1
55 kDa
40 kDa
WB: β-actin
D
100 kDa
70 kDa
WB: MORC2
55 kDa
40 kDa
WB: PGK1
100 kDa
70 kDa
WB: MORC2
55 kDa
40 kDa
WB: PGK1
55 kDa
40 kDa
WB: β-actin
E
55 kDa
40 kDa
WB: CK2
55 kDa
40 kDa
WB: PGK1
55 kDa
40 kDa
WB: CK2
55 kDa
40 kDa
WB: PGK1
55 kDa
40 kDa
WB: β-actin
F
55 kDa
40 kDa
WB: PGK1 pS256
55 kDa
40 kDa
WB: PGK1
55 kDa
40 kDa
WB: β-actin
G
130 kDa
100 kDa
WB: MORC2
55 kDa
40 kDa
WB: PGK1
55 kDa
40 kDa
WB: PGK1
130 kDa
100 kDa
WB: MORC2
40 kDa
35 kDa
WB: β-PGK1
40 kDa
35 kDa
WB: β-actin

Fig. 4

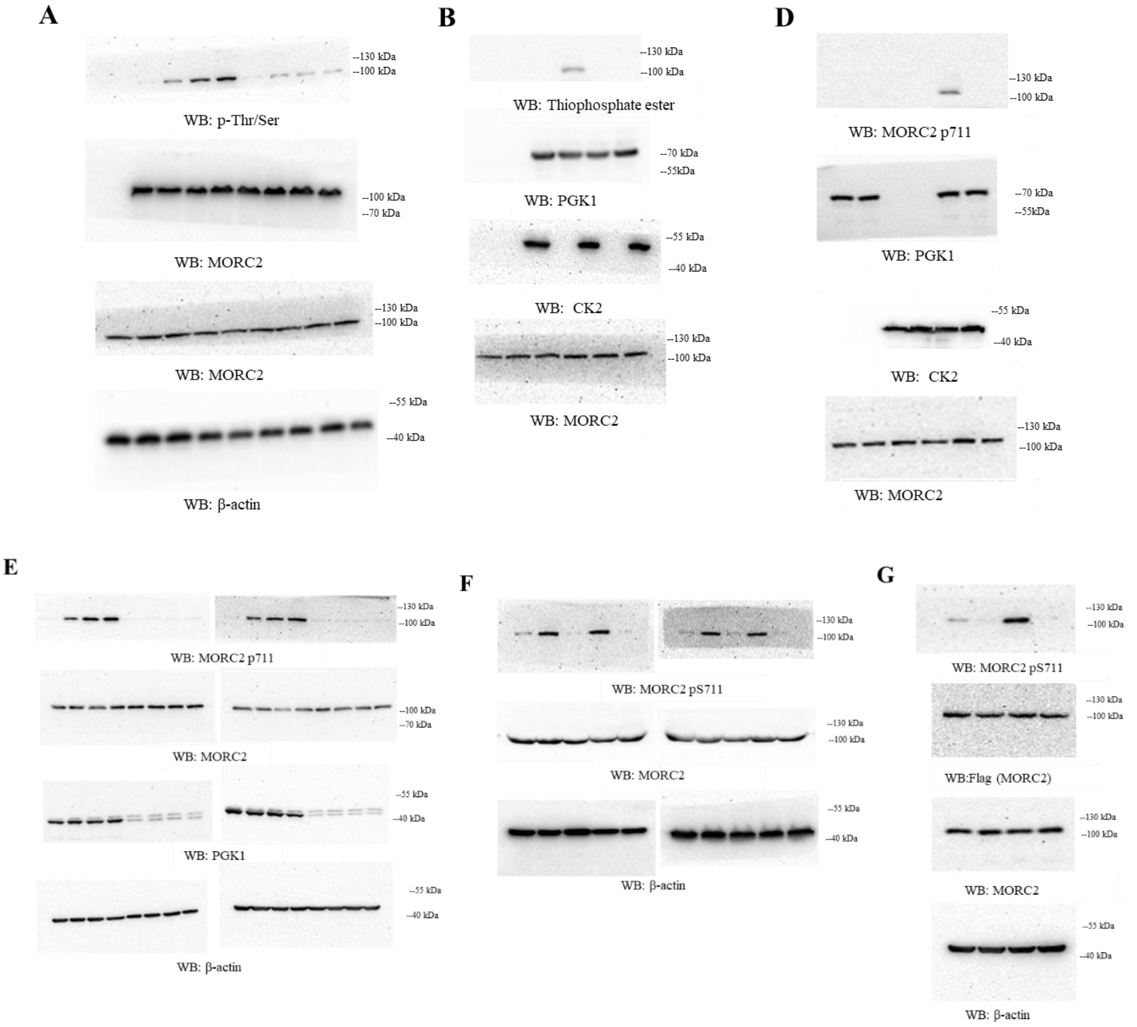
A
--130 kDa
--100 kDa
WB: p-Thr/Ser
--100 kDa
--70 kDa
WB: MORC2
--130 kDa
--100 kDa
WB: MORC2
--55 kDa
--40 kDa
WB: β-actin
B
--130 kDa
--100 kDa
WB: Thiophosphate ester
--70 kDa
--55kDa
WB: PGK1
--55 kDa
--40 kDa
WB: CK2
--130 kDa
--100 kDa
WB: MORC2
D
--130 kDa
--100 kDa
WB: MORC2 p711
--70 kDa
--55kDa
WB: PGK1
--55 kDa
--40 kDa
WB: CK2
--130 kDa
--100 kDa
WB: MORC2
E
--130 kDa
--100 kDa
WB: MORC2 p711
--100 kDa
--70 kDa
WB: MORC2
--55 kDa
--40 kDa
WB: PGK1
--55 kDa
--40 kDa
WB: β-actin
F
--130 kDa
--100 kDa
WB: MORC2 pS711
--130 kDa
--100 kDa
WB: MORC2
--55 kDa
--40 kDa
WB: β-actin
G
--130 kDa
--100 kDa
WB: MORC2 pS711
--130 kDa
--100 kDa
WB:Flag (MORC2)
--130 kDa
--100 kDa
WB: MORC2
--55 kDa
--40 kDa
WB: β-actin

Fig. 5

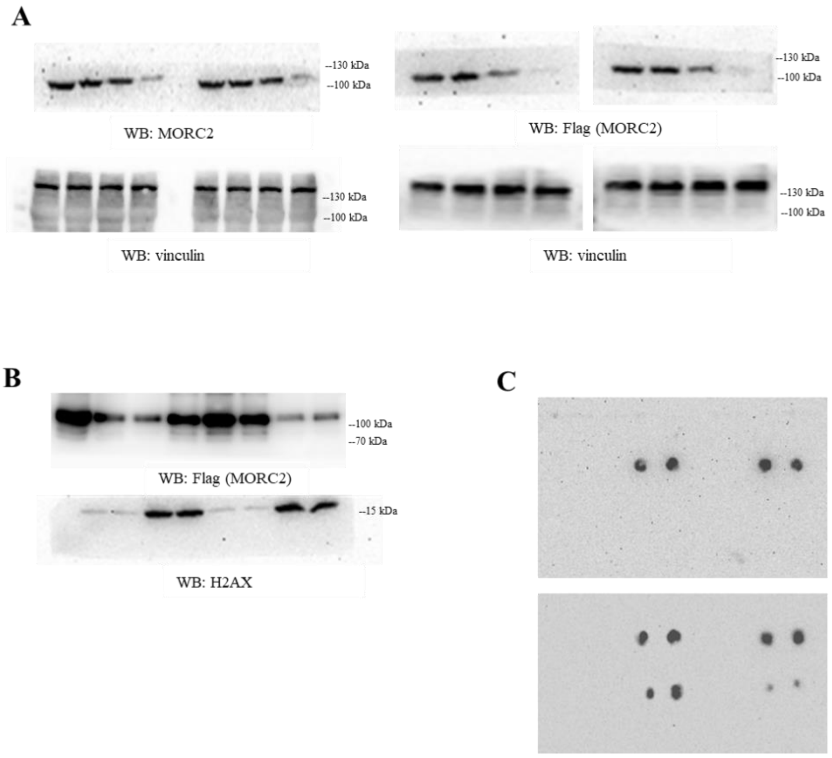
A
--130 kDa
--100 kDa
WB: MORC2
--130 kDa
--100 kDa
WB: Flag (MORC2)
--130 kDa
--100 kDa
WB: vinculin
--130 kDa
--100 kDa
WB: vinculin
B
--100 kDa
--70 kDa
WB: Flag (MORC2)
--15 kDa
WB: H2AX
C

SF. 3

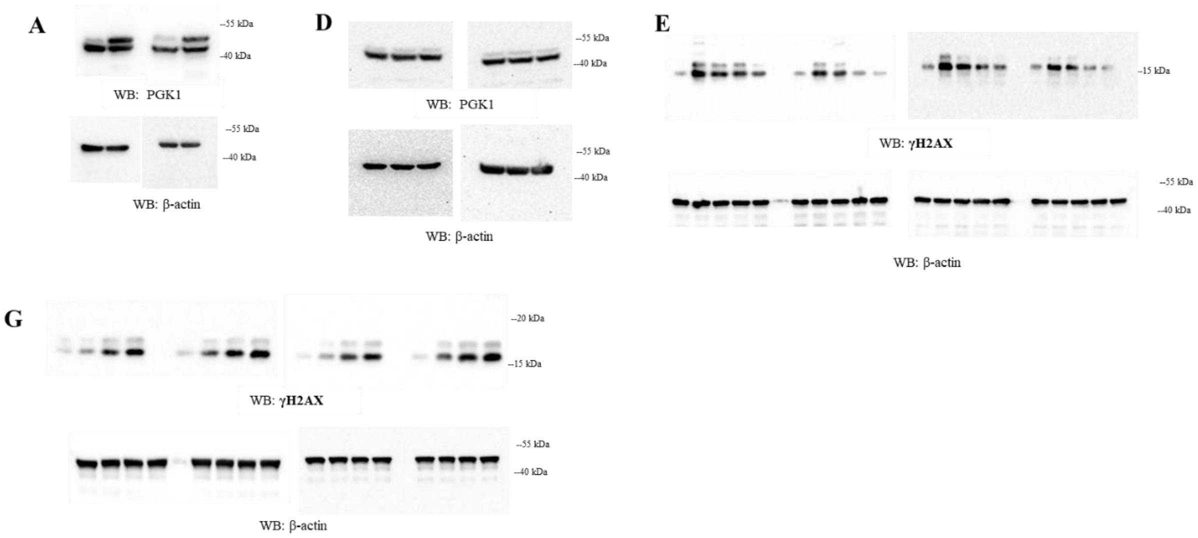
A
--55 kDa
--40 kDa
WB: PGK1
--55 kDa
--40 kDa
WB: β-actin
D
--55 kDa
--40 kDa
WB: PGK1
--55 kDa
--40 kDa
WB: β-actin
E
--15 kDa
WB: γH2AX
--55 kDa
--40 kDa
WB: β-actin
G
--20 kDa
--15 kDa
WB: γH2AX
--55 kDa
--40 kDa
WB: β-actin

SF. 4

A

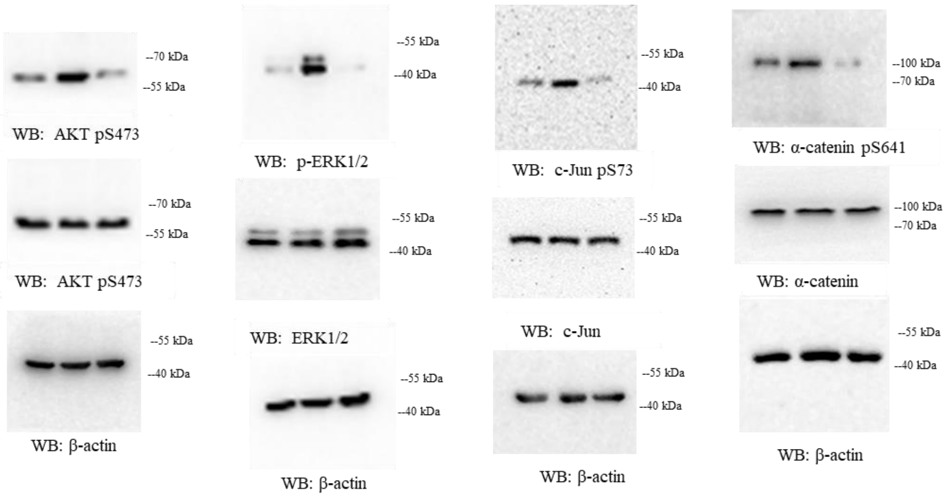

70 kDa
55 kDa
WB: AKT pS473
70 kDa
55 kDa
WB: AKT pS473
55 kDa
40 kDa
WB: β-actin
55 kDa
40 kDa
WB: p-ERK1/2
55 kDa
40 kDa
WB: ERK1/2
55 kDa
40 kDa
WB: β-actin
55 kDa
40 kDa
WB: c-Jun pS73
55 kDa
40 kDa
WB: c-Jun
55 kDa
40 kDa
WB: β-actin
100 kDa
70 kDa
WB: α-catenin pS641
100 kDa
70 kDa
WB: α-catenin
55 kDa
40 kDa
WB: β-actin

SF. 5

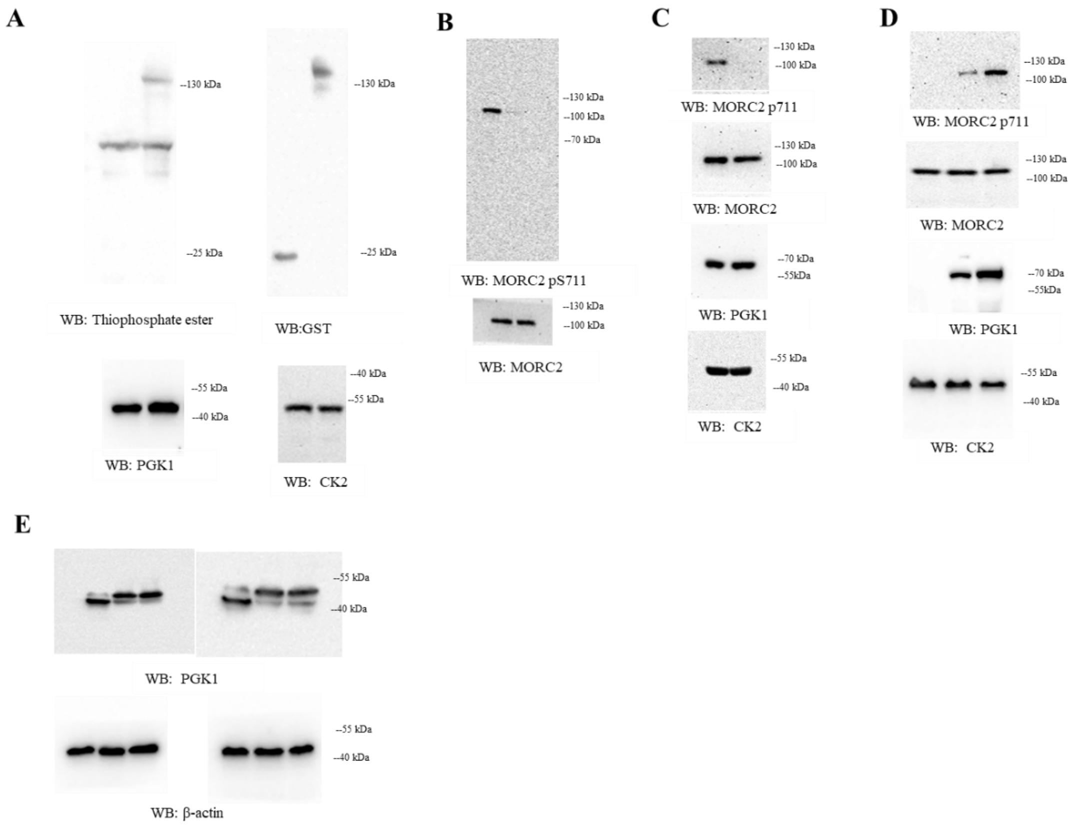
A
WB: Thiophosphate ester
WB:GST
WB: PGK1
WB: CK2
B
WB: MORC2 pS711
WB: MORC2
C
WB: MORC2 p711
WB: MORC2
WB: PGK1
WB: CK2
D
WB: MORC2 p711
WB: MORC2
WB: PGK1
WB: CK2
E
WB: PGK1
WB: β-actin
130 kDa
100 kDa
70 kDa
55 kDa
40 kDa
25 kDa

SF. 6

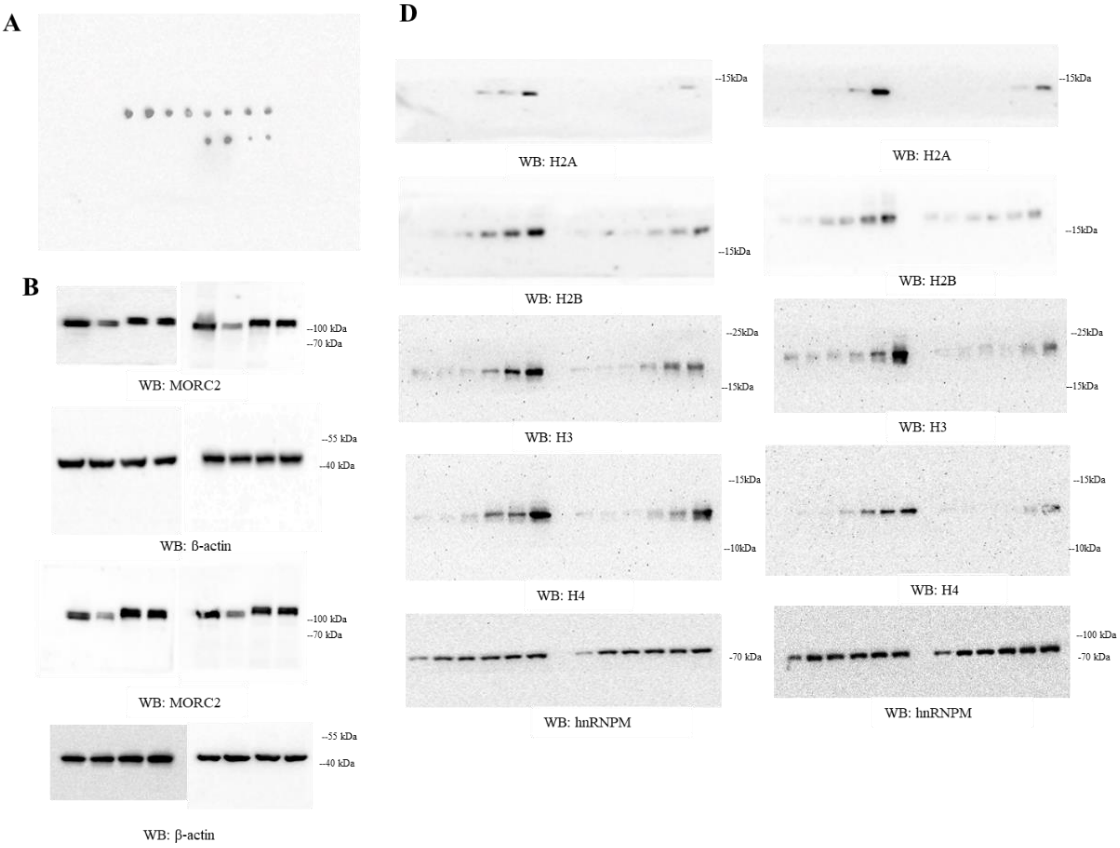
A
B
WB: MORC2
100 kDa
70 kDa
WB: β-actin
55 kDa
40 kDa
WB: MORC2
WB: β-actin
D
15kDa
WB: H2A
WB: H2A
WB: H2B
WB: H2B
25kDa
WB: H3
WB: H3
10kDa
WB: H4
WB: H4
70 kDa
WB: hnRNPM
WB: hnRNPM

SF. 8

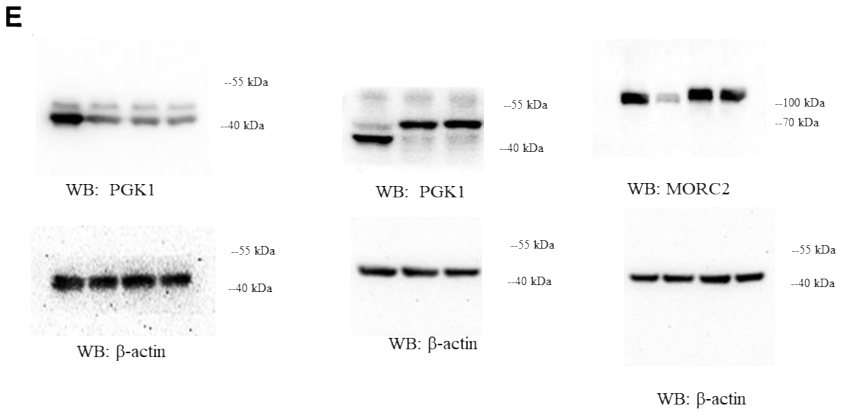
E
--55 kDa
--40 kDa
WB: PGK1
--55 kDa
--40 kDa
WB: PGK1
--100 kDa
--70 kDa
WB: MORC2
--55 kDa
--40 kDa
WB: β-actin
--55 kDa
--40 kDa
WB: β-actin
--55 kDa
--40 kDa
WB: β-actin
